# Obesity reduces hippocampal structure and function in older African Americans with the APOE-ε4 Alzheimer’s disease risk allele

**DOI:** 10.3389/fnagi.2023.1239727

**Published:** 2023-09-04

**Authors:** Zuzanna Osiecka, Bernadette A. Fausto, Joshua L. Gills, Neha Sinha, Steven K. Malin, Mark A. Gluck

**Affiliations:** ^1^Aging and Brain Health Alliance, Center for Molecular and Behavioral Neuroscience, Rutgers University–Newark, Newark, NJ, United States; ^2^Department of Kinesiology and Health, School of Arts and Sciences, Rutgers University, New Brunswick, NJ, United States

**Keywords:** body mass index, dementia, medial temporal lobe, underrepresented populations, cognitive function

## Abstract

**Introduction:**

Excess body weight and Alzheimer’s disease (AD) disproportionately affect older African Americans. While mid-life obesity increases risk for AD, few data exist on the relationship between late-life obesity and AD, or how obesity-based and genetic risk for AD interact. Although the APOE-ε4 allele confers a strong genetic risk for AD, it is unclear if late-life obesity poses a greater risk for APOE-ε4 carriers compared to non-carriers. Here we assessed: (1) the influence of body mass index (BMI) (normal; overweight; class 1 obese; ≥ class 2 obese) on cognitive and structural MRI measures of AD risk; and (2) the interaction between BMI and APOE-ε4 in older African Americans.

**Methods:**

Seventy cognitively normal older African American participants (M_*age*_ = 69.50 years; M_*BMI*_ = 31.01 kg/m^2^; 39% APOE-ε4 allele carriers; 86% female) completed anthropometric measurements, physical assessments, saliva collection for APOE-ε4 genotyping, cognitive testing, health and lifestyle questionnaires, and structural neuroimaging [volume/surface area (SA) for medial temporal lobe subregions and hippocampal subfields]. Covariates included age, sex, education, literacy, depressive symptomology, and estimated aerobic fitness.

**Results:**

Using ANCOVAs, we observed that individuals who were overweight demonstrated better hippocampal cognitive function (generalization of learning: a sensitive marker of preclinical AD) than individuals with normal BMI, *p* = 0.016, η_*p*_2 = 0.18. However, individuals in the obese categories who were APOE-ε4 non-carriers had larger hippocampal subfield cornu Ammonis region 1 (CA1) volumes, while those who were APOE-ε4 carriers had smaller CA1 volumes, *p* = 0.003, η_*p*_2 = 0.23.

**Discussion:**

Thus, being overweight by BMI standards may preserve hippocampal function, but obesity reduces hippocampal structure and function in older African Americans with the APOE-ε4 Alzheimer’s disease risk allele.

## 1. Introduction

A burgeoning global health concern, obesity has nearly tripled since 1975 (Gerontological Society of America, 2021; WHO, 2021). Adults with obesity are at increased risk for numerous comorbidities, including cardiovascular diseases, stroke, type 2 diabetes, hypertension, and some cancers (CDC, 2021). Further, obesity is linked to increased Alzheimer’s disease (AD) risk, another growing public health crisis. Several studies highlight that obesity, independent of cardiovascular disease comorbidities, is a risk factor for later-life dementia (Whitmer et al., 2008). However, these findings mainly apply to obesity in *midlife* (Whitmer et al., 2008; Anstey et al., 2011; Xu et al., 2011; Meng et al., 2014), with relatively few studies demonstrating that late-life obesity is associated with increased AD risk (Gustafson et al., 2003). Conversely, several studies demonstrate that in later-life, obesity is associated with a lower risk of dementia (Atti et al., 2008; Fitzpatrick et al., 2009). Overall, while midlife obesity appears to be a risk factor for AD, later-life obesity may be neutral or even protective with respect to AD (Bischof and Park, 2015).

Similarly, whether late-life obesity is related to better or worse cognitive function remains equivocal. For example, while some studies indicate that late-life obesity is associated with poorer executive function, verbal abilities, and processing speed (Ihle et al., 2016; Yang et al., 2018), there is also evidence that late-life obesity may be associated with better cognitive function (Tolppanen et al., 2014). One study suggests that obesity in late life is positively associated with reasoning and speed of processing (Kuo et al., 2006). In a longitudinal study, late-life obesity was associated with attenuated decline in global cognition, perceptual speed, visuospatial ability, as well as episodic, semantic, and working memory (Aiken-Morgan et al., 2020). Thus, obesity may confer detrimental or protective effects to cognition as measured by standardized neuropsychological tests in later life.

Another area of consideration linking obesity and cognitive function or AD risk is related to brain atrophy and structural changes. In fact, some studies report higher body weight is associated with lower whole brain volume in middle-age (Enzinger et al., 2005; Ward et al., 2005; Taki et al., 2008). A 24-year follow-up study among older females reported that higher body mass index was related to greater temporal atrophy but there were no associations with frontal, parietal or occipital atrophy (Gustafson et al., 2004). However, most studies have examined whole brain or hippocampal volume, and more recent research suggests fine-scale subregional analysis of the hippocampus may have advantages over overall volume (e.g., see Apostolova et al., 2010; Younes et al., 2014). While the mechanisms linking obesity and brain atrophy are not fully understood, obesity is related to other risk factors associated with brain atrophy including reduced aerobic fitness (Colcombe et al., 2003), hyperlipidemia (Bruce-Keller et al., 2009), increased inflammation (Arnoldussen et al., 2014), and impaired respiratory function (Zammit et al., 2010).

Alarmingly, African Americans bear a disproportionate burden of both obesity and AD (US Department of Health and Human Services, 2020). Across the lifespan, African Americans are 1.4 times more likely to have obesity relative to non-Hispanic white Americans (US Department of Health and Human Services, 2020), and more than 80% of African American females are overweight or obese (National Center for Health Statistics, 2019). Notably, the prevalence of obesity in midlife is higher in African Americans (46.2%) than in non-Hispanic white Americans (34.9%) (Nianogo et al., 2022). In parallel, the lifetime prevalence of AD is two to three times higher among older African Americans than in older non-Hispanic whites (Rajan et al., 2019). Importantly, 21.7% of dementia cases among African Americans are attributable to mid-life obesity as compared to 17.3% of dementia cases in non-Hispanic whites (Nianogo et al., 2022). Although mid-life obesity increases risk for AD, we still know too little about the relationship between late-life obesity and AD, especially in older African Americans.

Previous research demonstrates that the relationship between cognitive function and **BMI** in older adults varies by race/ethnicity (Bryant et al., 2014). Both non-**Hispanic** whites and African Americans showed better cognitive function as **BMI** increased, but African Americans showed poorer cognition at extreme **BMI** levels (i.e., very low and very high). Notably, **BMI** standards may be too crude a measure to accurately classify older African Americans and other non-white samples, especially since body composition indices were derived from predominantly non-**Hispanic** white samples (Smalley et al., 1990; Wagner and Heyward, 2000). For example, recent research demonstrates that whereas black women have the highest levels of subcutaneous abdominal fat, **Hispanic** women have the highest levels of visceral abdominal fat (Banack et al., 2023); but **BMI** does not differentiate types of fat nor where they accumulate. Further research is necessary to determine how cognitive function associated with standard **BMI** cutoffs, particularly in the high or low **BMI** ranges, may vary ***within*** high-risk populations, such as older African Americans. In addition, other factors that vary by race/ethnicity may influence the obesity-cognition and obesity-brain structure relationships.

One possible factor confounding these relationships is AD genetic predisposition. The apolipoprotein (APOE) ε4 allele located on chromosome 19 is the most prevalent and well-studied genetic risk factor for late-onset AD (Rajan et al., 2014). Although African Americans are 1.4 times more likely to carry the APOE-ε4 allele than European Americans (Rajan et al., 2014), its effect on AD development is diminished in African ancestry groups (Hendrie et al., 2014; Rajabli et al., 2018). Still, African Americans with at least one copy of the APOE-ε4 allele are almost three times more likely to develop AD than their noncarrier counterparts (Farrer et al., 1997). Recent research, however, suggests environmental or health factors, such as obesity, may modify the influence of APOE-ε4 in some racial and ethnic groups (Rajabli et al., 2018). Indeed, in several studies among homozygotic twin pairs, environmental factors account for up to 20% in vulnerability to AD development, suggesting a gene-environment interaction (for a review, see Plassman and Breitner, 1996). Further, genetic factors account for about 50% of AD risk (Gatz et al., 2006, for a review, see Tang and Gershon, 2022). However, no work, to date, has systematically examined the interactions of obesity and AD risk via APOE-ε4 in older African American adults.

Therefore, the purpose of this study was to examine: (1) the influence of body mass index (BMI) groups (normal; overweight; class 1 obese; ≥ class 2 obese) on cognitive and structural MRI measures of AD risk; and (2) the interaction between BMI and APOE-ε4 in older African Americans. The current analyses focused on a novel cognitive outcome, generalization, the ability to apply previously learned rules to new situations and contexts (Myers et al., 2002). Previous studies have shown that generalization is a cognitive marker of AD risk with deficits preceding explicit memory declines (Myers et al., 2002, 2008; Myers and Ahmed, 2019). For structural neuroimaging, we examined the medial temporal lobe (MTL), including the hippocampus, given its particular vulnerability to AD in preclinical stages (Jagust et al., 2006). Further, both higher body weight and APOE-ε4 allele presence individually have been associated with smaller MTL region volumes among non-Hispanic white American and European older adult samples (Reiman et al., 1998; Gustafson et al., 2004; Cacciaglia et al., 2018).

## 2. Materials and methods

### 2.1. Participants

Participants were drawn from the database of an ongoing longitudinal study at Rutgers University–Newark. *Pathways to Healthy Aging in African Americans*. The *Pathways* study investigates links between cognition, health, lifestyle, genetic, brain structure and function, and sociodemographic variables of African Americans ages 60 and older. Notably, this age criterion is lower than the American Medical Association’s older adult age designation of 65+ due to life expectancy of African Americans estimated to be about 5 years lower than their white American counterparts (Hill et al., 2022). Participants were recruited through the *Aging and Brain Health Alliance*, a university-community partnership established in 2006 comprising community members from senior centers, public and subsidized housing, churches, health and wellness companies, and other organizations that serve greater Newark residents. Recruitment methods are detailed elsewhere (Gluck et al., 2018).

Participants included had to: (1) be ages 60 years or older; (2) identify as black or African American; (3) have intact mental status with a mini-mental state examination (MMSE) score of 24 (to exclude individuals with cognitive impairment including possible dementia); (4) have undergone neuroimaging; and (5) have provided a saliva sample for APOE genotyping analyses as part of the *Pathways* study. Exclusion criteria were: (1) diagnosis of any neurodegenerative disorders; taking medications typically prescribed for dementia such as Razadyne, Aricept, or Namenda; diagnosis of a learning disability; (2) self-reported excessive alcohol and/or recreational drug use; (3) had a medical procedure that required general anesthesia in the past 3 months; (4) presence of MRI contraindications (e.g., metallic stent, cardiac pacemaker, claustrophobia); (5) inability to see a computer screen at normal viewing distance; and (6) color blindness (because some of the cognitive tasks used colors as discriminative cues). Seventy participants met inclusion criteria for the present analyses and did not significantly differ from the larger *Pathways* study cohort participants (*N* = 404 participants at the time of data extraction) on age, sex composition, education, literacy, depressive symptomology, estimated aerobic fitness, or BMI, *p*s > 0.05. All participants provided written informed consent at enrollment. Ethical approval was granted by the Rutgers University institutional review board.

### 2.2. Procedure

Candidates were telephone screened to determine initial eligibility. Those potentially eligible attended an in-person screening at Rutgers University–Newark. After providing informed consent, participants who passed the in-person color-blindness and mental status screening proceeded with a full laboratory visit which consisted of cognitive assessments, physical function tests, anthropometric assessments, and health/lifestyle questionnaires. Participants provided a saliva sample for APOE genotyping and returned for a neuroimaging visit within 2 weeks of the initial laboratory visit.

### 2.3. Measures

#### 2.3.1. Body mass index

Body mass index (BMI) was used as an estimate of body fat and computed by dividing weight (in kilograms, kg) by height (in meters, m). Participants were categorized into four groups per the American Association of Clinical Endocrinologists clinical practice guidelines (Garvey et al., 2016):

(1) Normal weight: BMI of 18.5 to 24.9 kg/m^2^;

(2) Overweight: BMI of 25 kg/m^2^ to 29.9 kg/m^2^;

(3) Class 1 obese: 30.0 to 34.9 kg/m^2^ and;

(4) Class 2 obese or greater BMI > 35.0 kg/m^2^.

#### 2.3.2. APOE-ε 4

Saliva samples were collected using Oragene kits for DNA extraction. Before processing for data analyses, saliva sample vials were kept in a secure storage room in the laboratory. Biosafety-trained research staff transported the samples to an offsite biotechnology facility for genotyping. Genotyping was conducted via quantitative PCR on an Eppendorf Mastercycler, using a TaqMan custom genotyping assay. Following genotyping, participants were characterized as either APOE-ε4 *non*-carriers or APOE-ε4 carriers, regardless of hetero- or homozygosity as described before (Foster et al., 2017). Importantly, we retained all APOE-ε4 heterozygotes including individuals with ε2/ε4 genotype given that the mere presence of ε4 confers greater risk for AD (Liu et al., 2013).

#### 2.3.3. Cognition

##### 2.3.3.1. Rey auditory verbal learning test (RAVLT)

As a word learning test, RAVLT is used to assess episodic memory (Rey, 1964). Participants are presented with a list of 15 words and asked to immediately recall as many words from that list as possible. This process is repeated a total of five consecutive times. Participants are then presented with a new list of 15 words (distractor list) and asked to immediately recall as many words from the distractor list as possible. They are then immediately asked to recall as many words from the first word list. After a 30-min delay, participants are once again asked to recall as many words from the first list. The scores obtained for this task include the sum of the first five trials to assess total learning recall, the sum of items recalled after the distractor list is presented (short delay recall), and the sum of items recalled after a 30-min time lapse (long delay recall).

##### 2.3.3.2. Concurrent Discrimination and Transfer Task

**FIGURE 1:**
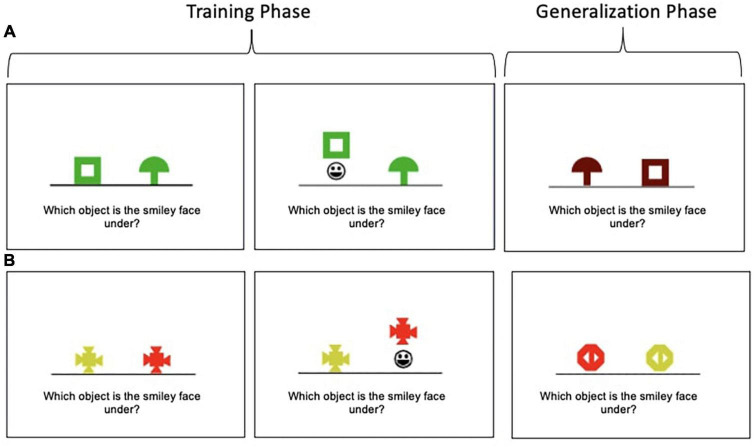
Concurrent Discrimination and Transfer Task examples. **(A)** In this example, participants learn to choose the green box in preference to the green mushroom during the training phase. Thus, shape is predictive (relevant), but color is not (irrelevant). In the generalization phase, participants are presented with a brown box and brown mushroom; the brown box is the correct object; thus, shape is still predictive, but the irrelevant feature (color) has been altered. **(B)** In this example, color is predictive (relevant), but shape is not (irrelevant).

The Concurrent Discrimination and Transfer Task assesses the ability to generalize past learning to novel situations and has been shown to be sensitive to subtle changes in the hippocampus and MTL circuits implicated in preclinical AD (Myers et al., 2008, 2002). The task involves two phases: (1) a training phase during which participants learn to discriminate between the incorrect and correct object in eight object pairs based on trial-to-trial feedback and (2) a generalization phase during which participants apply previously learned information to discriminate between the incorrect and correct object among novel object pairs. Example trials of both phases are depicted in Figure 1 and more details on the task are published elsewhere (Myers and Ahmed, 2019). Performance is recorded as the number of generalization phase errors committed; lower scores indicate better performance.

### 2.4. Hippocampal subfield and medial temporal lobe region volumetry

#### 2.4.1. MRI data acquisition

Structural imaging data were acquired from a Siemens Trio 3T MRI scanner equipped with 32-channel Multiband parallel encoding head coils using T1-weighted 3-dimensional high-resolution magnetization prepared rapid acquisition with gradient echo (i.e., MP-RAGE) sequence in sagittal plane with the following parameters: repetition time/echo time/inversion time = 2,300/2.95/900 ms; flip angle 9°; resolution 1.1 mm × 1.1 mm × 1.2 mm; 176 contiguous slices acquired sagittally; field of view = 270 × 254 × 212. Scans were visually inspected by a neuroimaging specialist in order to ensure appropriate data quality.

#### 2.4.2. MRI analysis

Advanced Normalization Tools (ANTs; Klein et al., 2009) was used to account for global brain shape, including intracranial volume and size differences for all participants by normalizing all MPRAGE scans to an in-house high-resolution 0.65 mm isotropic custom template. **ANT**s implements a powerful diffeomorphic registration algorithm known as symmetric normalization (**SyN**). Each participant’s scan and regions of interest (**ROI**) segmentation map were used to warp gray/white matter boundary to the gray/cerebrospinal fluid boundary into the custom template space. Further, **SyN** uses the inter-boundary distance to estimate thickness within specified anatomical constraints. The following subregions of the **MTL** and hippocampal subfields were segmented bilaterally: perirhinal cortex (**PRC**), parahippocampal cortex (**PHC**), posteromedial entorhinal cortex (**pMEC**), anterolateral EC (**aLEC**), subiculum, cornu **Ammonis** region 1 (CA1), and dentate gyrus/CA3 (DG/CA3). **ROI**s were segmented based on published protocols (Reagh et al., 2018). The DG/CA3 were combined into one label as per previously published protocols (Yassa et al., 2010). Anterolateral and posteromedial entorhinal cortex were segmented based on results from Maass et al. (2015), as also applied by Reagh et al. (2018). Previous hippocampal morphology studies in cognitively intact individuals at genetic risk for **AD** found a detrimental effect of APOE-ε 4 on CA1 and DG/CA3 subfield thickness (for a review, see de Flores et al., 2015). Evidence of this focal atrophy in at-risk individuals underscores hippocampal and **MTL** segmentation as a promising technique in **AD**-related research.

### 2.5. Covariates

Age, sex, education, literacy level [North American Adult Reading Test errors (NAART); Uttl, 2002], depressive symptomology [Beck Depression Inventory-II (BDI-II); Beck et al., 1996] and estimated aerobic fitness (derived from the Six-Minute Walk Test; Steffen et al., 2002, Ross et al., 2010) were included as covariates as per previous studies (Fausto and Gluck, 2020). We estimated participants’ maximal oxygen consumption (i.e., VO_2_max estimate) using the following formula: VO_2_max estimate = [4.948+(0.023*Distance walked in meters)] (Ross et al., 2010).

### 2.6. Statistical analysis

One-way analyses of covariance (ANCOVAs) were used to compare the BMI groups, while controlling for age, sex, education, literacy, depressive symptomology, APOE-ε4 status, and estimated aerobic fitness. We also conducted a series of 2 × 2 between-groups ANCOVAs to test for BMI APOE-ε4 status interactions on all cognitive and structural imaging outcomes while controlling for age, sex, education, literacy, depressive symptomology, and estimated aerobic fitness. Intracranial volume was added as a covariate for all structural imaging outcomes. The Benjamini and Hochberg (1995) step up procedure was used to control the false discovery rate (FDR) for simultaneous testing of multiple independent hypotheses.

### 2.7. Power analysis

A power analysis using G*Power software version 3.1.9.6 (Faul et al., 2009) determined that a sample size of 70 has at least 80% power to detect a medium effect size for a significant main effect of BMI group on cognitive and structural imaging outcomes (α = 0.05, two-tailed). The sample size of 70 also has more than 80% power to detect a medium effect size for significant BMI × APOE-ε4 status interactions (α = 0.05, two-tailed) with six groups [four BMI groups (normal, overweight, class 1 obese, class 2 obese) and two APOE-ε4 status groups (ε4 carrier, non-carrier)].

## 3. Results

Descriptive statistics for the 70 participants are included in Table 1. Participants were ages 60 to 90 years and completed, on average, an associate’s degree or some college coursework. The vast majority of the participants were female (86%). Almost half (47%) of the participants’ BMI fell in the obese category and 39% were APOE-ε4 allele carriers. See Table 2 for volumes and SAs by BMI group. See Supplementary Data Sheet 1, 2 for complete dataset and statistical coding, respectively.

**TABLE 1:** Descriptive statistics of analytic sample (*N* = 70).

Variable	Normal BMI (18.5–24.9) (*n* = 13)		Overweight BMI (25.0–29.9) (*n* = 24)		Class 1 obese BMI (30.0–34.9) (*n* = 16)		Class 2 obese BMI (≥ 35.0) (*n* = 17)	
	Mean (*n*)	SD (%)	Mean (*n*)	SD (%)	Mean (*n*)	SD (%)	Mean (*n*)	SD (%)
Age	70.77	8.97	69.08	5.88	71.44	9.15	67.29	4.86
Sex (female)	(11)	(84.60)	(18)	(75.00)	(15)	(93.80)	(16)	(94.10)
Education (years)	13.19	0.88	14.31	2.17	13.78	1.96	14.88	2.39
NAART (errors)	13.15	11.41	34.96	11.87	41.81	8.63	36.12	12.30
BDI-II (0–63 points)	7.08	5.97	6.54	5.30	10.38	7.15	8.59	5.67
VO_2_max Est. (mL/kg/min)	16.15	2.42	15.54	2.57	14.31	2.43	14.13	1.96
APOE-ε4 allele (present)	(6)	(46.20)	(8)	(33.30)	(6)	(37.50)	(7)	(42.20)
**RAVLT**								
Learning	40.77	11.09	41.08	9.62	38.19	5.39	43.41	8.49
Short delay	6.77	3.49	7.29	2.51	6.94	2.62	7.47	3.45
Long delay	6.62	4.31	7.13	2.92	7.00	2.78	7.59	3.55
Concurrent Discrimination and Transfer Task (errors)	19.77	20.99	6.50	12.31	9.69	12.96	10.76	16.81

MMSE, mini-mental state examination; NAART, North American Adult Reading Test; BDI-II, Beck Depression Inventory-II; BMI, body mass index in kg/m^2^; RAVLT, Rey auditory verbal learning test. Est., estimate.

**TABLE 2:** Volumes and surface areas (mm^2^) for structural imaging outcomes by body mass index group (*N* = 70).

Variable	Normal BMI (18.5–24.9) (*n* = 13)		Overweight BMI (25.0–29.9) (*n* = 24)		Class 1 obese BMI (30.0–34.9) (*n* = 16)		Class 2 obese BMI (≥ 35.0) (*n* = 17)	
Structural outcome (mm^3^)	Mean (*n*)	SD (%)	Mean (*n*)	SD (%)	Mean (*n*)	SD (%)	Mean (*n*)	SD (%)
Volume left aLEC	443.07	64.96	460.58	94.20	419.98	59.87	415.06	61.65
SA left aLEC	442.48	53.33	459.65	65.73	423.14	47.66	428.73	46.99
Volume right aLEC	471.30	62.80	486.44	80.44	458.19	71.08	412.07	58.54
SA right aLEC	462.39	37.57	477.53	52.54	454.49	51.62	426.33	47.83
Volume left pMEC	256.08	44.02	282.32	45.33	267.64	43.39	269.17	55.66
SA left pMEC	311.45	35.73	326.39	41.48	324.97	34.86	311.92	46.04
Volume right pMEC	328.89	34.25	344.45	56.72	326.04	51.41	313.30	70.08
SA right pMEC	370.15	27.54	378.22	46.08	362.17	40.21	349.02	54.79
Volume left PRC	1,098.17	347.29	1,226.72	382.91	981.03	206.25	957.87	241.24
SA left PRC	739.56	155.83	794.84	154.97	684.86	100.56	672.64	116.37
Volume right PRC	1,109.26	295.04	1,148.02	279.40	905.86	214.48	983.94	266.77
SA right PRC	773.35	134.77	765.11	102.30	657.49	111.32	695.54	120.11
Volume left PHC	2,377.87	412.81	2,383.92	302.95	2,241.79	256.63	2,158.05	265.95
SA left PHC	1,417.31	125.24	1,419.93	124.18	1,338.80	111.00	1,302.33	125.98
V right PHC	2,272.42	360.69	2,259.78	308.95	2,053.41	300.77	2,156.01	274.53
SA right PHC	1,359.34	129.16	1,366.01	124.46	1,272.28	106.23	1,287.34	112.63
V left DG/CA3	534.14	95.86	589.83	84.35	569.14	55.33	545.32	91.73
SA left DG/CA3	565.16	70.42	599.75	61.40	592.71	50.71	578.11	71.04
V right DG/CA3	640.11	91.04	673.93	99.17	637.64	74.00	615.29	101.64
SA right DG/CA3	640.15	73.10	657.71	64.62	632.98	52.16	616.12	66.60
V left CA1	1,441.32	150.55	1,582.96	161.48	1,495.19	148.83	1,525.81	167.63
SA left CA1	1,331.57	120.78	1,415.63	99.73	1,397.27	126.50	1,404.25	106.79
V right CA1	1,475.52	143.18	1,628.01	169.65	1,504.95	179.95	1,551.64	158.05
SA right CA1	1,351.39	104.70	1,447.66	103.83	1,388.42	135.79	1,413.58	95.43
V left subiculum	638.77	71.31	702.68	108.36	698.12	73.51	699.54	85.87
SA left subiculum	695.83	46.15	725.52	78.60	739.87	65.02	748.53	59.75
V right subiculum	623.48	55.20	709.53	102.16	674.26	57.12	691.45	80.49
SA right subiculum	649.03	35.60	713.46	68.74	699.02	53.09	710.05	59.06

aLEC, anterolateral entorhinal cortex; pMEC, posteromedial entorhinal cortex; PRC, perirhinal cortex; PHC, parahippocampal cortex; DG/CA3 = dentate gyrus/CA3.

### 3.1. Cognitive outcomes

#### 3.1.1. Main effects of BMI on cognitive outcomes

**FIGURE 2:**
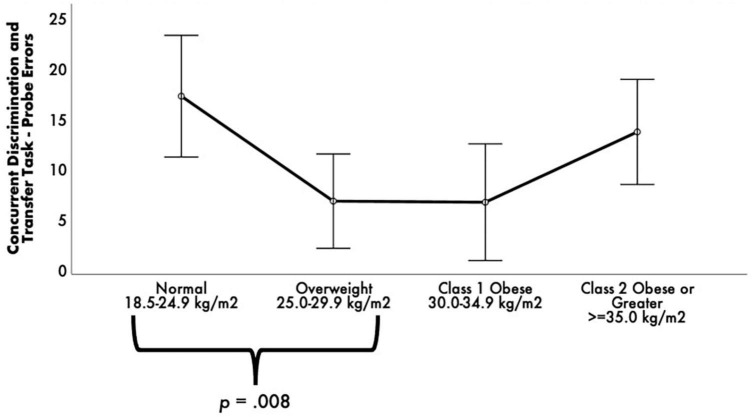
Estimated marginal means of Concurrent Discrimination and Transfer Task errors by body mass index (BMI) groups. ANCOVA revealed a statistically significant difference between BMI groups on Concurrent Discrimination and Transfer Task–Probe Errors. *Post-hoc* pairwise comparisons showed that the normal group committed significantly greater Probe Errors than the overweight and obese groups. Covariates appearing in the model are evaluated at the following values: age = 68.99, education = 14.16, sex 0 = F, l = M = 0.1493, depressive symptomology (Beck Depression Inventory-II) = 8.03, literacy (NAART) = 36.24, Concurrent Discrimination and Transfer Task–training errors = 23.24, Vo2max estimate = 15.04. Error bars: +/– 2 SE.

While there were no statistically significant differences between BMI groups on RAVLT performance, *p*s > 0.05, FDR-corrected *p*s = not significant. Concurrent Discrimination and Transfer Task–Probe Errors was different among BMI groups [*F*(3, 52) = 3.76, *p* = 0.016, FDR-corrected *p*_*critical*_ = 0.02, η_*p*_^2^ = 0.18] (see Figure 2). Post-hoc comparisons revealed that the normal group committed significantly greater Probe Errors than the overweight group, *p* = 0.008, FDR-corrected *p*_*critical*_ = 0.008.

#### 3.1.2. BMI ×APOE-ε4 status interactions on cognitive outcomes

Separate ANCOVAs were also conducted to test interactions between BMI group and APOE-ε4 status on cognitive outcomes. There were no significant BMI ×APOE-ε4 status interactions on any cognitive outcomes before and after covariate adjustment, *p*s > 0.05, FDR-corrected *p*s = not significant.

### 3.2. Structural imaging outcomes

#### 3.2.1. Main effects of BMI on structural imaging outcomes

Although there were no significant main effects for BMI on any structural imaging outcomes before and after covariate adjustment, the following regions/subfields showed trending main effects that did not survive FDR correction: right aLEC volume (*p* = 0.012, FDR-corrected *p*_*critical*_ = 0.004); right at volume (*p* = 0.021, FDR-corrected *p*_*critical*_ = 0.007); left CA1 volume (*p* = 0.042, FDR-corrected *p*_*critical*_ = 0.01); and right aLEC surface area (*p* = 0.024, FDR-corrected *p*_*critical*_ = 0.011).

#### 3.2.2. BMI ×APOE-ε4 status interactions on imaging outcomes

**FIGURE 3:**
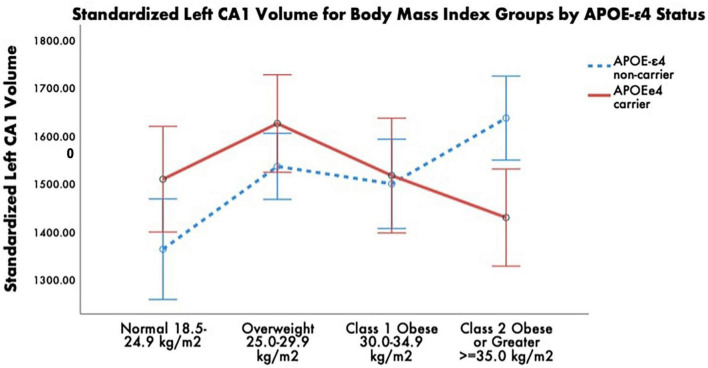
Estimated marginal means of standardized left CA1 volume for body mass index (BMI) groups by APOE-ε4 status. ANCOVA revealed a statistically significant BMI × APOE-ε4 status interaction. Within the APOE-ε4 non-carriers, *post-hoc* pairwise comparisons revealed that the normal BMI group had significantly smaller left CA1 volumes than the overweight and class 2/severe obesity groups. There was no significant main effect of BMI group within the APOE-ε4 carriers. Covariates appearing in the model are evaluated at the following values: age = 68.99, education = 14.16, sex 0 = F, l = M = 0.1493, depressive symptomology (Beck Depression Inventory-II) = 8.03, Literacy (NAART) = 36.24, VO_2_max estimate = 15.04, ICV = 1,339,302.4607. Error bars: +/– 2 SE.

There was, nonetheless, a significant BMI group ×APOE-ε4 status interaction for left CA1 volume, *F*(3, 52) = 5.02, *p* = 0.004, FDR-corrected *p*_*critical*_ = 0.007, η_*p*_^2^ = 0.23 (see Figure 3). BMI (*p* = 0.042) and intracranial volume (*p* = 0.005) emerged as significant covariates. The significant interaction was followed by examining APOE-ε4 non-carriers and carriers separately with post-hoc one-way ANCOVAs among the four BMI groups. For the APOE-ε4 non-carriers, there was a significant main effect of BMI group, *F*(3, 31) = 7.79, *p* < 0.001, η_p_^2^ = 0.43. Post-hoc pairwise comparisons revealed that the normal BMI group had significantly smaller left CA1 volumes than the overweight and class 2/severe obesity groups, *p* < 0.001, FDR-corrected *p*s_critical_ < 0.02. For the APOE-ε4 carriers, there was no significant main effect of BMI group, *F*(3, 14) = 0.98, *p* = 0.43, η_p_^2^ = 0.17. Exploratory post-hoc pairwise comparisons revealed that the overweight BMI group had larger left CA1 volumes than the class 2/severity obesity group, with this contrast approaching significance, *p* = 0.12, FDR-corrected *p*s_critical_ = 0.04.

## 4. Discussion

We examined the independent effects of BMI group and the interactive effects of BMI group and APOE-ε4 status on MTL/hippocampal cognitive function and structure among older African Americans. There were two main findings: (1) older African Americans in the overweight range demonstrate better ability to apply previously learned rules to novel situations (i.e., generalization performance) than individuals in the normal BMI range; and (2) among older African Americans who are APOE-ε4 non-carriers, obesity is associated with larger CA1 volumes. In contrast, among APOE-ε4 carriers, obesity is associated with smaller CA1 volumes. Overall, these data suggest being overweight by BMI standards may preserve hippocampal function, but obesity reduces hippocampal structure and function in older African Americans with the APOE- ε4 Alzheimer’s disease risk allele.

Among older adults, conflicting studies have reported overweight or obesity in late life to be both detrimental and beneficial to cognitive function (Han et al., 2009, Walther et al., 2010). Our study findings support being overweight but not obese in late life may be beneficial to hippocampal-dependent cognitive performance. This is consistent with prior work across older white, African, and Hispanic Americans showing these BMI ranges to be protective for global cognitive function (Kuo et al., 2006; Yoon et al., 2012; Bryant et al., 2014) as well as for reasoning, processing speed, and memory (Kuo et al., 2006). Our study, though, is the first to identify that excess body weight is neuroprotective for generalization performance in older African Americans, independent of their demographics and other potential confounding variables (i.e., fitness levels, depressive symptomology). These findings suggest standard BMI cutoffs for being overweight may systematically overestimate adiposity and its negative effects on cognition and AD risk in this population. Some evidence suggests alternative anthropometric measures of central obesity (e.g., waist-to-hip ratio) may be better predictors of cardiovascular and cognitive outcomes than BMI (Luchsinger et al., 2012). Given reports that body morphology/composition differs by race and ethnicity, future studies should compare the relative contributions of BMI and measures of central obesity to cognitive function and AD development within at-risk populations such as older African Americans.

Importantly, while our study examined cross-sectional associations, longitudinal changes in BMI appear to contribute to dementia risk (Kadey et al., 2021; Guo et al., 2022). One study of a predominantly non-Hispanic white sample (84.2%) found that among only APOE-ε4 allele carriers, a decline in BMI over 5 years was associated with conversion to mild cognitive impairment or dementia (Kadey et al., 2021). Similarly, in another non-Hispanic white majority sample (85.6% white), individuals who went on to develop mild cognitive impairment had lower BMIs about 7 years preceding their diagnosis as compared to their cognitively unimpaired counterparts (Guo et al., 2022). The decreases in BMI as a risk factor for cognitive decline could be attributed to negative health consequences that accompany obesity such as chronic inflammation and muscle atrophy (as a result of reduced physical activity and/or aerobic fitness). Indeed, the current study cohort showed low overall estimated aerobic fitness levels (see Table 1; Mean overall VO_2_max estimate = 15.04 mL/kg/min) which is a lower reported estimate than other groups that analyzed older adults samples (e.g., Mean VO2max estimates 19.1 and 22.8 mL/kg/min for women and men, respectively; Paterson et al., 1999). Lower BMIs preceding cognitive impairment may be attributed to alterations in appetite regulation that may influence hippocampal plasticity and hence cognitive performance and status (Fadel et al., 2013). Together, BMI declines in later life (and related maladaptive health behaviors) along with a genetic predisposition may increase risk for dementia. Future studies should examine how the interactions of late-life body weight changes and genetic risk factors (e.g., APOE-ε4) influence cognitive change and status over time within underrepresented populations in biomedical research, including older African Americans.

Our study also observed that obesity effects on hippocampal subfield volume may differ by APOE-ε4 carrier status. Specifically, APOE-ε4 non-carriers had larger left CA1 subfields if they were in the overweight or higher ranges of BMI relative to individuals in the normal BMI range. However, APOE-ε4 carriers had smaller CA1 subfields if they were in the obese range relative to individuals in the overweight range. The differential effect of AD genetic risk on CA1 volume suggests late-life obesity is neuroprotective for APOE-ε4 non-carriers while particularly detrimental to brain health for APOE-ε4 carriers.

Although a recent cross-sectional study found that older European American individuals with higher BMI and high genetic risk for AD had smaller hippocampal and entorhinal cortex volumes (Hayes et al., 2020), the present study is the first to identify, via fine-scale subregion structural analysis, that BMI and APOE-ε4 synergistically impact CA1 volume specifically. Indeed, the CA1 is particularly susceptible to Alzheimer’s pathology early in the disease course (Zarow et al., 2005; de Flores et al., 2015), and atrophy in this subfield is linked to poorer verbal memory and global cognition (Csernansky et al., 2005; Apostolova et al., 2006). That genetic and health risk factors (i.e., obesity) synergistically affect CA1 subfield volume may help explain why African Americans–who have higher prevalence of both the APOE-ε4 allele and obesity–are more likely to develop AD. The mechanism by which obesity interacts with genetic factors is beyond the scope of this work, but obesity has been suggested to be neuroprotective due to hormonal changes (e.g., leptin, estrogen), dietary factors (e.g., vitamin E and D levels), and less risk for fragility (Yaffe et al., 2000; Holden et al., 2009; Bove et al., 2013).

### 4.1. Limitations

The study has considerations that warrant mentioning. This work is limited to cross-sectional data. Therefore, directionality of the observed relationships cannot be determined. Further, given the findings and prior evidence of differences in body morphology by race (Wagner and Heyward, 2000), BMI may overestimate morbidity and negative cognitive health consequences in older African Americans. Indeed, the American Medical Association recently voted to adopt a policy to move away from using BMI as a standalone measure of weight and health (Blum, 2023). Other measures such as waist circumference, waist-to-hip ratio, and body composition metrics via dual x-ray absorptiometry or CT/MRI scans of the abdomen to discern total body fat, abdominal fat, and fat-free mass may be better indicators of excess weight and correlates of health morbidity in this population (Flint et al., 2010).

Further, there is a growing appreciation that metabolic conditions, such as obesity, and exposures across the lifespan including differences in access to education as well as varied literacy levels, stress, and socioeconomic circumstances may further contribute to disparities in cognitive health (Barnes and Bennett, 2014). Some of these factors were outside the scope of the current study (we controlled for age, sex, education, literacy, depressive symptomology, and estimated aerobic fitness). However, future studies may consider adding such factors to analyses.

Recent reviews purport that the ∼1 mm^3^ resolution for T1-weighted images may be insufficient to visualize hippocampal subfield structure (Bussy et al., 2021; Wisse et al., 2021). Nevertheless, similar to our findings, several studies have consistently demonstrated the CA1 subfield to be the first impacted in the AD development trajectory despite varying resolution parameters (Bussy et al., 2021). Indeed, it will be important to better examine these BMI-genetic-neuroimaging relationships using higher resolution scanning in future investigations.

Finally, our sample comprised almost 86% older African American females; thus, findings may only be generalizable in this subgroup. Sensitivity analyses conducted on the subset of females (*n* = 60) revealed similar trends of neuroprotection by obesity and negative synergistic effects of BMI APOE-ε4 on left CA1 volume. Further, prior research suggests that equivocal findings on the BMI-cognition relationships may be due to sex-specific differences, with overweight and obesity being more neuroprotective among females than males (Armstrong et al., 2019). Future studies should recruit higher proportions of males in order to test for sex differences in the examination of BMI and BMI ×APOE-ε4 interactions on MTL cognitive function and structure.

## 5. Conclusion

Being overweight (relative to normal weight by BMI standards) may be neuroprotective with respect to MTL/hippocampal-dependent cognitive function in older African Americans. However, in the obese BMI ranges (30 kg/m^2^), AD genetic susceptibility conferred by the APOE-ε4 allele may compound the risk for hippocampal atrophy in this population. Future studies should investigate the potential overlap between the metabolic sequelae of late-life obesity and genetic mechanisms underlying AD. The present data highlight the importance of examining the synergistic effects of health and genetic risk factors on markers for AD in diverse populations with the ultimate goal of developing ways to delay progression to AD.

## Data availability statement

The original contributions presented in this study are included in the article/Supplementary materials, further inquiries can be directed to the corresponding authors.

## Ethics statement

The studies involving humans were approved by the Rutgers University Institutional Review Board. The studies were conducted in accordance with the local legislation and institutional requirements. The participants provided their written informed consent to participate in this study.

## Author contributions

ZO, BF, and MG contributed to conception and design of the study. ZO and BF organized the database, performed the statistical analysis, and wrote the first draft of the manuscript. NS, JG, SM, and MG wrote sections of the manuscript. All authors read and revised the manuscript and approved the submitted version.
