# Supplementary material for: Obesity reduces hippocampal structure and function in older African Americans with the APOE-ε4 Alzheimer’s disease risk allele
Source: Front Aging Neurosci. 2023 Sep 4;15:1239727. doi: 10.3389/fnagi.2023.1239727 (PMC10507275; doi:10.3389/fnagi.2023.1239727)
Supplement: Supplementary file 2 [file Data_Sheet_2.PDF]

Statistical Code for Manuscript "Obesity reduces hippocampal structure and function in older African Americans with the APOE-4 Alzheimer's disease risk allele"

```
* Encoding: UTF-8.
***NOV 30 2020*****

*************BMIxAPOEe4 interactions on MTL structure and
cognition*************************
**********************Data prep and analysis syntax*************************


******Checking for univariate outliers******

DATASET ACTIVATE DataSet1.
DESCRIPTIVES VARIABLES=Age Education_yrs BDI_Total NAART_Total BMI ChooseProbenErr
FGen
  RAVLT_Recall_Total_A1A5 RAVLT_Recall_Total_A6 RAVLT_Recall_Total_A7 VLeftaLEC
SALeftaLEC VRightaLEC
  SARightaLEC VLeftpMEC SALeftpMEC VRightpMEC SARightpMEC VLeftPerirhinalCortex
  SALeftPerirhinalCortex VRightPerirhinalCortex SARightPerirhinalCortex
VLeftParahippocampalCortex
  SALeftParahippocampalCortex VRightParahippocampalCortex SARightParahippocampalCortex
VLeftDGCA3
  SALeftDGCA3 VRightDGCA3 SARightDGCA3 VLeftCA1 SALeftCA1 VRightCA1 SARightCA1
VLeftSubiculum
  SALeftSubiculum VRightSubiculum SARightSubiculum
 /SAVE
 /STATISTICS=MEAN STDDEV MIN MAX.

********Checking for multivariate outliers using Mahalanobis distance computation*******

REGRESSION
 /MISSING LISTWISE
 /STATISTICS COEFF OUTS R ANOVA
 /CRITERIA=PIN(.05) POUT(.10)
 /NOORIGIN
 /DEPENDENT VLeftDGCA3
 /METHOD=ENTER Age Education_yrs BDI_Total NAART_Total GENDER_USE BMI APOE_USE
ChooseTrainingnErr
 /SAVE MAHAL.

COMPUTE Probability_MAH_1=1-CDF.CHISQ(MAH_1, 8).
EXECUTE.
```

```
COMPUTE
MAH_outlier = (Probability_MAH_1 < .001).
EXECUTE.

****No multivariate outliers******

**************************Re-analyze using BMI categories of obesity********
https://www.cdc.gov/obesity/adult/defining.html

RECODE BMI (Lowest thru 24.9=0) (25 thru 29.9=1) (30 thru 34.9=2) (35 thru Highest=3) INTO
BMI4grp.
VARIABLE LABELS  BMI4grp 'BMI four group: 0= <24.9; 1=25-29.9; 2=30-34.9; 3=>35'.
EXECUTE.

**********Demographics for analytic sample N=70***************

DESCRIPTIVES VARIABLES=Age Education_yrs BDI_Total NAART_Total VO2 BMI log10BMI
RAVLT_Recall_Total_A1A5
    RAVLT_Recall_Total_A6 RAVLT_Recall_Total_A7 LearnMemCOMP FGen ChooseProbenErr
ChooseTrainingnErr
    Flexibility_March  VLeftaLEC SALeftaLEC VRightaLEC SARightaLEC VLeftpMEC SALeftpMEC
VRightpMEC SARightpMEC
    VLeftPerirhinalCortex SALeftPerirhinalCortex VRightPerirhinalCortex SARightPerirhinalCortex
    VLeftParahippocampalCortex SALeftParahippocampalCortex VRightParahippocampalCortex
    SARightParahippocampalCortex VLeftDGCA3 SALeftDGCA3 VRightDGCA3 SARightDGCA3
VLeftCA1 SALeftCA1
    VRightCA1 SARightCA1 VLeftSubiculum SALeftSubiculum VRightSubiculum SARightSubiculum
  /STATISTICS=MEAN STDDEV MIN MAX.

FREQUENCIES VARIABLES=BMIgroup  BMI4grp APOE_USE GENDER_USE
  /ORDER=ANALYSIS.

*************************COGNITIVE
OUTCOMES*************************************
    *******Feb 3, 2021- add VO2 as additional covariate*******

******1) Choose Probe Errors******

UNIANOVA ChooseProbenErr BY BMI4grp APOE_USE WITH Age Education_yrs GENDER_USE
BDI_Total
    NAART_Total ChooseTrainingnErr VO2
  /CONTRAST(BMI4grp)=Repeated
  /CONTRAST(APOE_USE)=Simple(1)
```

```
  /METHOD=SSTYPE(3)
  /INTERCEPT=INCLUDE
  /PLOT=PROFILE(BMI4grp*APOE_USE BMI4grp) TYPE=LINE ERRORBAR=SE(2)
MEANREFERENCE=NO YAXIS=AUTO
  /EMMEANS=TABLES(OVERALL) WITH(Age=MEAN Education_yrs=MEAN GENDER_USE=MEAN
BDI_Total=MEAN
    NAART_Total=MEAN ChooseTrainingnErr=MEAN VO2=MEAN)
  /EMMEANS=TABLES(BMI4grp) WITH(Age=MEAN Education_yrs=MEAN GENDER_USE=MEAN
BDI_Total=MEAN
    NAART_Total=MEAN ChooseTrainingnErr=MEAN VO2=MEAN) COMPARE ADJ(LSD)
  /EMMEANS=TABLES(APOE_USE) WITH(Age=MEAN Education_yrs=MEAN GENDER_USE=MEAN
BDI_Total=MEAN
    NAART_Total=MEAN ChooseTrainingnErr=MEAN VO2=MEAN) COMPARE ADJ(LSD)
  /EMMEANS=TABLES(APOE_USE*BMI4grp) WITH(Age=MEAN Education_yrs=MEAN
GENDER_USE=MEAN BDI_Total=MEAN
    NAART_Total=MEAN ChooseTrainingnErr=MEAN VO2=MEAN)
  /PRINT ETASQ DESCRIPTIVE PARAMETER HOMOGENEITY OPOWER
  /CRITERIA=ALPHA(.05)
  /DESIGN=BMI4grp APOE_USE Age Education_yrs GENDER_USE BDI_Total NAART_Total
ChooseTrainingnErr VO2
    APOE_USE*BMI4grp.

*********2) Fish Generalization**********

UNIANOVA  FGen BY BMI4grp APOE_USE WITH Age Education_yrs GENDER_USE BDI_Total VO2
    NAART_Total
  /CONTRAST(BMI4grp)=Repeated
  /CONTRAST(APOE_USE)=Simple(1)
  /METHOD=SSTYPE(3)
  /INTERCEPT=INCLUDE
  /PLOT=PROFILE(BMI4grp*APOE_USE BMI4grp) TYPE=LINE ERRORBAR=SE(2)
MEANREFERENCE=NO YAXIS=AUTO
  /EMMEANS=TABLES(OVERALL) WITH(Age=MEAN Education_yrs=MEAN GENDER_USE=MEAN
BDI_Total=MEAN
    NAART_Total=MEAN VO2=MEAN)
  /EMMEANS=TABLES(BMI4grp) WITH(Age=MEAN Education_yrs=MEAN GENDER_USE=MEAN
BDI_Total=MEAN
    NAART_Total=MEAN VO2=MEAN) COMPARE ADJ(LSD)
  /EMMEANS=TABLES(APOE_USE) WITH(Age=MEAN Education_yrs=MEAN GENDER_USE=MEAN
BDI_Total=MEAN
    NAART_Total=MEAN VO2=MEAN) COMPARE ADJ(LSD)
  /EMMEANS=TABLES(APOE_USE*BMI4grp) WITH(Age=MEAN Education_yrs=MEAN
GENDER_USE=MEAN BDI_Total=MEAN
    NAART_Total=MEAN VO2=MEAN)
```

```
  /PRINT ETASQ DESCRIPTIVE PARAMETER HOMOGENEITY OPOWER
  /CRITERIA=ALPHA(.05)
  /DESIGN=BMI4grp APOE_USE Age Education_yrs GENDER_USE BDI_Total NAART_Total VO2
    APOE_USE*BMI4grp.

***********3) RAVLT Learning***************

UNIANOVA  RAVLT_Recall_Total_A1A5 BY BMI4grp APOE_USE WITH Age Education_yrs
GENDER_USE BDI_Total VO2
    NAART_Total
  /CONTRAST(BMI4grp)=Repeated
  /CONTRAST(APOE_USE)=Simple(1)
  /METHOD=SSTYPE(3)
  /INTERCEPT=INCLUDE
  /PLOT=PROFILE(BMI4grp*APOE_USE BMI4grp) TYPE=LINE ERRORBAR=SE(2)
MEANREFERENCE=NO YAXIS=AUTO
  /EMMEANS=TABLES(OVERALL) WITH(Age=MEAN Education_yrs=MEAN GENDER_USE=MEAN
BDI_Total=MEAN
    NAART_Total=MEAN VO2=MEAN)
  /EMMEANS=TABLES(BMI4grp) WITH(Age=MEAN Education_yrs=MEAN GENDER_USE=MEAN
BDI_Total=MEAN
    NAART_Total=MEAN VO2=MEAN) COMPARE ADJ(LSD)
  /EMMEANS=TABLES(APOE_USE) WITH(Age=MEAN Education_yrs=MEAN GENDER_USE=MEAN
BDI_Total=MEAN
    NAART_Total=MEAN VO2=MEAN) COMPARE ADJ(LSD)
  /EMMEANS=TABLES(APOE_USE*BMI4grp) WITH(Age=MEAN Education_yrs=MEAN
GENDER_USE=MEAN BDI_Total=MEAN
    NAART_Total=MEAN VO2=MEAN)
  /PRINT ETASQ DESCRIPTIVE PARAMETER HOMOGENEITY OPOWER
  /CRITERIA=ALPHA(.05)
  /DESIGN=BMI4grp APOE_USE Age Education_yrs GENDER_USE BDI_Total NAART_Total VO2
    APOE_USE*BMI4grp.

************4) RAVLT Short Delay*****************

UNIANOVA  RAVLT_Recall_Total_A6 BY BMI4grp APOE_USE WITH Age Education_yrs
GENDER_USE BDI_Total VO2
    NAART_Total
  /CONTRAST(BMI4grp)=Repeated
  /CONTRAST(APOE_USE)=Simple(1)
  /METHOD=SSTYPE(3)
  /INTERCEPT=INCLUDE
  /PLOT=PROFILE(BMI4grp*APOE_USE BMI4grp) TYPE=LINE ERRORBAR=SE(2)
MEANREFERENCE=NO YAXIS=AUTO
```

```
  /EMMEANS=TABLES(OVERALL) WITH(Age=MEAN Education_yrs=MEAN GENDER_USE=MEAN
BDI_Total=MEAN
    NAART_Total=MEAN VO2=MEAN)
  /EMMEANS=TABLES(BMI4grp) WITH(Age=MEAN Education_yrs=MEAN GENDER_USE=MEAN
BDI_Total=MEAN
    NAART_Total=MEAN VO2=MEAN) COMPARE ADJ(LSD)
  /EMMEANS=TABLES(APOE_USE) WITH(Age=MEAN Education_yrs=MEAN GENDER_USE=MEAN
BDI_Total=MEAN
    NAART_Total=MEAN VO2=MEAN) COMPARE ADJ(LSD)
  /EMMEANS=TABLES(APOE_USE*BMI4grp) WITH(Age=MEAN Education_yrs=MEAN
GENDER_USE=MEAN BDI_Total=MEAN
    NAART_Total=MEAN VO2=MEAN)
  /PRINT ETASQ DESCRIPTIVE PARAMETER HOMOGENEITY OPOWER
  /CRITERIA=ALPHA(.05)
  /DESIGN=BMI4grp APOE_USE Age Education_yrs GENDER_USE BDI_Total NAART_Total VO2
    APOE_USE*BMI4grp.
```

*************5) RAVLT Long Delay******************

```
UNIANOVA   RAVLT_Recall_Total_A7 BY BMI4grp APOE_USE WITH Age Education_yrs
GENDER_USE BDI_Total VO2
    NAART_Total
  /CONTRAST(BMI4grp)=Repeated
  /CONTRAST(APOE_USE)=Simple(1)
  /METHOD=SSTYPE(3)
  /INTERCEPT=INCLUDE
  /PLOT=PROFILE(BMI4grp*APOE_USE BMI4grp) TYPE=LINE ERRORBAR=SE(2)
MEANREFERENCE=NO YAXIS=AUTO
  /EMMEANS=TABLES(OVERALL) WITH(Age=MEAN Education_yrs=MEAN GENDER_USE=MEAN
BDI_Total=MEAN
    NAART_Total=MEAN VO2=MEAN)
  /EMMEANS=TABLES(BMI4grp) WITH(Age=MEAN Education_yrs=MEAN GENDER_USE=MEAN
BDI_Total=MEAN
    NAART_Total=MEAN VO2=MEAN) COMPARE ADJ(LSD)
  /EMMEANS=TABLES(APOE_USE) WITH(Age=MEAN Education_yrs=MEAN GENDER_USE=MEAN
BDI_Total=MEAN
    NAART_Total=MEAN VO2=MEAN) COMPARE ADJ(LSD)
  /EMMEANS=TABLES(APOE_USE*BMI4grp) WITH(Age=MEAN Education_yrs=MEAN
GENDER_USE=MEAN BDI_Total=MEAN
    NAART_Total=MEAN VO2=MEAN)
  /PRINT ETASQ DESCRIPTIVE PARAMETER HOMOGENEITY OPOWER
  /CRITERIA=ALPHA(.05)
  /DESIGN=BMI4grp APOE_USE Age Education_yrs GENDER_USE BDI_Total NAART_Total VO2
```

```
   APOE_USE*BMI4grp.

************************************STRUCTURAL IMAGING
OUTCOMES************************************

*******1) Left Anterolateral entorhinal cortex**********

UNIANOVA   VLeftaLEC BY BMI4grp APOE_USE WITH Age Education_yrs GENDER_USE BDI_Total
VO2
   NAART_Total
  /CONTRAST(BMI4grp)=Repeated
  /CONTRAST(APOE_USE)=Simple(1)
  /METHOD=SSTYPE(3)
  /INTERCEPT=INCLUDE
  /PLOT=PROFILE(BMI4grp*APOE_USE BMI4grp) TYPE=LINE ERRORBAR=SE(2)
MEANREFERENCE=NO YAXIS=AUTO
  /EMMEANS=TABLES(OVERALL) WITH(Age=MEAN Education_yrs=MEAN GENDER_USE=MEAN
BDI_Total=MEAN
   NAART_Total=MEAN VO2=MEAN)
  /EMMEANS=TABLES(BMI4grp) WITH(Age=MEAN Education_yrs=MEAN GENDER_USE=MEAN
BDI_Total=MEAN
   NAART_Total=MEAN VO2=MEAN) COMPARE ADJ(LSD)
  /EMMEANS=TABLES(APOE_USE) WITH(Age=MEAN Education_yrs=MEAN GENDER_USE=MEAN
BDI_Total=MEAN
   NAART_Total=MEAN VO2=MEAN) COMPARE ADJ(LSD)
  /EMMEANS=TABLES(APOE_USE*BMI4grp) WITH(Age=MEAN Education_yrs=MEAN
GENDER_USE=MEAN BDI_Total=MEAN
   NAART_Total=MEAN VO2=MEAN)
  /PRINT ETASQ DESCRIPTIVE PARAMETER HOMOGENEITY OPOWER
  /CRITERIA=ALPHA(.05)
  /DESIGN=BMI4grp APOE_USE Age Education_yrs GENDER_USE BDI_Total NAART_Total VO2
   APOE_USE*BMI4grp.

UNIANOVA   SALeftaLEC BY BMI4grp APOE_USE WITH Age Education_yrs GENDER_USE
BDI_Total VO2
   NAART_Total
  /CONTRAST(BMI4grp)=Repeated
  /CONTRAST(APOE_USE)=Simple(1)
  /METHOD=SSTYPE(3)
  /INTERCEPT=INCLUDE
  /PLOT=PROFILE(BMI4grp*APOE_USE BMI4grp) TYPE=LINE ERRORBAR=SE(2)
MEANREFERENCE=NO YAXIS=AUTO
  /EMMEANS=TABLES(OVERALL) WITH(Age=MEAN Education_yrs=MEAN GENDER_USE=MEAN
BDI_Total=MEAN
```

```
    NAART_Total=MEAN VO2=MEAN)
  /EMMEANS=TABLES(BMI4grp) WITH(Age=MEAN Education_yrs=MEAN GENDER_USE=MEAN
BDI_Total=MEAN
    NAART_Total=MEAN VO2=MEAN) COMPARE ADJ(LSD)
  /EMMEANS=TABLES(APOE_USE) WITH(Age=MEAN Education_yrs=MEAN GENDER_USE=MEAN
BDI_Total=MEAN
    NAART_Total=MEAN VO2=MEAN) COMPARE ADJ(LSD)
  /EMMEANS=TABLES(APOE_USE*BMI4grp) WITH(Age=MEAN Education_yrs=MEAN
GENDER_USE=MEAN BDI_Total=MEAN
    NAART_Total=MEAN VO2=MEAN)
  /PRINT ETASQ DESCRIPTIVE PARAMETER HOMOGENEITY OPOWER
  /CRITERIA=ALPHA(.05)
  /DESIGN=BMI4grp APOE_USE Age Education_yrs GENDER_USE BDI_Total NAART_Total VO2
    APOE_USE*BMI4grp.

*********2) Right anterolateral entorhinal cortex***************

UNIANOVA  VRightaLEC BY BMI4grp APOE_USE WITH Age Education_yrs GENDER_USE
BDI_Total VO2
    NAART_Total
  /CONTRAST(BMI4grp)=Repeated
  /CONTRAST(APOE_USE)=Simple(1)
  /METHOD=SSTYPE(3)
  /INTERCEPT=INCLUDE
  /PLOT=PROFILE(BMI4grp*APOE_USE BMI4grp) TYPE=LINE ERRORBAR=SE(2)
MEANREFERENCE=NO YAXIS=AUTO
  /EMMEANS=TABLES(OVERALL) WITH(Age=MEAN Education_yrs=MEAN GENDER_USE=MEAN
BDI_Total=MEAN
    NAART_Total=MEAN VO2=MEAN)
  /EMMEANS=TABLES(BMI4grp) WITH(Age=MEAN Education_yrs=MEAN GENDER_USE=MEAN
BDI_Total=MEAN
    NAART_Total=MEAN VO2=MEAN) COMPARE ADJ(LSD)
  /EMMEANS=TABLES(APOE_USE) WITH(Age=MEAN Education_yrs=MEAN GENDER_USE=MEAN
BDI_Total=MEAN
    NAART_Total=MEAN VO2=MEAN) COMPARE ADJ(LSD)
  /EMMEANS=TABLES(APOE_USE*BMI4grp) WITH(Age=MEAN Education_yrs=MEAN
GENDER_USE=MEAN BDI_Total=MEAN
    NAART_Total=MEAN VO2=MEAN)
  /PRINT ETASQ DESCRIPTIVE PARAMETER HOMOGENEITY OPOWER
  /CRITERIA=ALPHA(.05)
  /DESIGN=BMI4grp APOE_USE Age Education_yrs GENDER_USE BDI_Total NAART_Total VO2
    APOE_USE*BMI4grp.
```

```
UNIANOVA  SARightaLEC BY BMI4grp APOE_USE WITH Age Education_yrs GENDER_USE
BDI_Total VO2
    NAART_Total
  /CONTRAST(BMI4grp)=Repeated
  /CONTRAST(APOE_USE)=Simple(1)
  /METHOD=SSTYPE(3)
  /INTERCEPT=INCLUDE
  /PLOT=PROFILE(BMI4grp*APOE_USE BMI4grp) TYPE=LINE ERRORBAR=SE(2)
MEANREFERENCE=NO YAXIS=AUTO
  /EMMEANS=TABLES(OVERALL) WITH(Age=MEAN Education_yrs=MEAN GENDER_USE=MEAN
BDI_Total=MEAN
    NAART_Total=MEAN VO2=MEAN)
  /EMMEANS=TABLES(BMI4grp) WITH(Age=MEAN Education_yrs=MEAN GENDER_USE=MEAN
BDI_Total=MEAN
    NAART_Total=MEAN VO2=MEAN) COMPARE ADJ(LSD)
  /EMMEANS=TABLES(APOE_USE) WITH(Age=MEAN Education_yrs=MEAN GENDER_USE=MEAN
BDI_Total=MEAN
    NAART_Total=MEAN VO2=MEAN) COMPARE ADJ(LSD)
  /EMMEANS=TABLES(APOE_USE*BMI4grp) WITH(Age=MEAN Education_yrs=MEAN
GENDER_USE=MEAN BDI_Total=MEAN
    NAART_Total=MEAN VO2=MEAN)
  /PRINT ETASQ DESCRIPTIVE PARAMETER HOMOGENEITY OPOWER
  /CRITERIA=ALPHA(.05)
  /DESIGN=BMI4grp APOE_USE Age Education_yrs GENDER_USE BDI_Total NAART_Total VO2
    APOE_USE*BMI4grp.
```

**********3) Left posteromedial entorhinal cortex*****************

```
UNIANOVA   VLeftpMEC BY BMI4grp APOE_USE WITH Age Education_yrs GENDER_USE
BDI_Total VO2
    NAART_Total
  /CONTRAST(BMI4grp)=Repeated
  /CONTRAST(APOE_USE)=Simple(1)
  /METHOD=SSTYPE(3)
  /INTERCEPT=INCLUDE
  /PLOT=PROFILE(BMI4grp*APOE_USE BMI4grp) TYPE=LINE ERRORBAR=SE(2)
MEANREFERENCE=NO YAXIS=AUTO
  /EMMEANS=TABLES(OVERALL) WITH(Age=MEAN Education_yrs=MEAN GENDER_USE=MEAN
BDI_Total=MEAN
    NAART_Total=MEAN VO2=MEAN)
  /EMMEANS=TABLES(BMI4grp) WITH(Age=MEAN Education_yrs=MEAN GENDER_USE=MEAN
BDI_Total=MEAN
    NAART_Total=MEAN VO2=MEAN) COMPARE ADJ(LSD)
```

```
 /EMMEANS=TABLES(APOE_USE) WITH(Age=MEAN Education_yrs=MEAN GENDER_USE=MEAN
BDI_Total=MEAN
   NAART_Total=MEAN VO2=MEAN) COMPARE ADJ(LSD)
 /EMMEANS=TABLES(APOE_USE*BMI4grp) WITH(Age=MEAN Education_yrs=MEAN
GENDER_USE=MEAN BDI_Total=MEAN
   NAART_Total=MEAN VO2=MEAN)
 /PRINT ETASQ DESCRIPTIVE PARAMETER HOMOGENEITY OPOWER
 /CRITERIA=ALPHA(.05)
 /DESIGN=BMI4grp APOE_USE Age Education_yrs GENDER_USE BDI_Total NAART_Total VO2
   APOE_USE*BMI4grp.

UNIANOVA   SALeftpMEC BY BMI4grp APOE_USE WITH Age Education_yrs GENDER_USE
BDI_Total VO2
   NAART_Total
 /CONTRAST(BMI4grp)=Repeated
 /CONTRAST(APOE_USE)=Simple(1)
 /METHOD=SSTYPE(3)
 /INTERCEPT=INCLUDE
 /PLOT=PROFILE(BMI4grp*APOE_USE BMI4grp) TYPE=LINE ERRORBAR=SE(2)
MEANREFERENCE=NO YAXIS=AUTO
 /EMMEANS=TABLES(OVERALL) WITH(Age=MEAN Education_yrs=MEAN GENDER_USE=MEAN
BDI_Total=MEAN
   NAART_Total=MEAN VO2=MEAN)
 /EMMEANS=TABLES(BMI4grp) WITH(Age=MEAN Education_yrs=MEAN GENDER_USE=MEAN
BDI_Total=MEAN
   NAART_Total=MEAN VO2=MEAN) COMPARE ADJ(LSD)
 /EMMEANS=TABLES(APOE_USE) WITH(Age=MEAN Education_yrs=MEAN GENDER_USE=MEAN
BDI_Total=MEAN
   NAART_Total=MEAN VO2=MEAN) COMPARE ADJ(LSD)
 /EMMEANS=TABLES(APOE_USE*BMI4grp) WITH(Age=MEAN Education_yrs=MEAN
GENDER_USE=MEAN BDI_Total=MEAN
   NAART_Total=MEAN VO2=MEAN)
 /PRINT ETASQ DESCRIPTIVE PARAMETER HOMOGENEITY OPOWER
 /CRITERIA=ALPHA(.05)
 /DESIGN=BMI4grp APOE_USE Age Education_yrs GENDER_USE BDI_Total NAART_Total VO2
   APOE_USE*BMI4grp.

******************4) Right posteromedial entorhinal cortex********

UNIANOVA   VRightpMEC BY BMI4grp APOE_USE WITH Age Education_yrs GENDER_USE
BDI_Total VO2
   NAART_Total
 /CONTRAST(BMI4grp)=Repeated
 /CONTRAST(APOE_USE)=Simple(1)
```

```
  /METHOD=SSTYPE(3)
  /INTERCEPT=INCLUDE
  /PLOT=PROFILE(BMI4grp*APOE_USE BMI4grp) TYPE=LINE ERRORBAR=SE(2)
MEANREFERENCE=NO YAXIS=AUTO
  /EMMEANS=TABLES(OVERALL) WITH(Age=MEAN Education_yrs=MEAN GENDER_USE=MEAN
BDI_Total=MEAN
    NAART_Total=MEAN VO2=MEAN)
  /EMMEANS=TABLES(BMI4grp) WITH(Age=MEAN Education_yrs=MEAN GENDER_USE=MEAN
BDI_Total=MEAN
    NAART_Total=MEAN VO2=MEAN) COMPARE ADJ(LSD)
  /EMMEANS=TABLES(APOE_USE) WITH(Age=MEAN Education_yrs=MEAN GENDER_USE=MEAN
BDI_Total=MEAN
    NAART_Total=MEAN VO2=MEAN) COMPARE ADJ(LSD)
  /EMMEANS=TABLES(APOE_USE*BMI4grp) WITH(Age=MEAN Education_yrs=MEAN
GENDER_USE=MEAN BDI_Total=MEAN
    NAART_Total=MEAN VO2=MEAN)
  /PRINT ETASQ DESCRIPTIVE PARAMETER HOMOGENEITY OPOWER
  /CRITERIA=ALPHA(.05)
  /DESIGN=BMI4grp APOE_USE Age Education_yrs GENDER_USE BDI_Total NAART_Total VO2
    APOE_USE*BMI4grp.


UNIANOVA   SARightpMEC BY BMI4grp APOE_USE WITH Age Education_yrs GENDER_USE
BDI_Total VO2
    NAART_Total
  /CONTRAST(BMI4grp)=Repeated
  /CONTRAST(APOE_USE)=Simple(1)
  /METHOD=SSTYPE(3)
  /INTERCEPT=INCLUDE
  /PLOT=PROFILE(BMI4grp*APOE_USE BMI4grp) TYPE=LINE ERRORBAR=SE(2)
MEANREFERENCE=NO YAXIS=AUTO
  /EMMEANS=TABLES(OVERALL) WITH(Age=MEAN Education_yrs=MEAN GENDER_USE=MEAN
BDI_Total=MEAN
    NAART_Total=MEAN VO2=MEAN)
  /EMMEANS=TABLES(BMI4grp) WITH(Age=MEAN Education_yrs=MEAN GENDER_USE=MEAN
BDI_Total=MEAN
    NAART_Total=MEAN VO2=MEAN) COMPARE ADJ(LSD)
  /EMMEANS=TABLES(APOE_USE) WITH(Age=MEAN Education_yrs=MEAN GENDER_USE=MEAN
BDI_Total=MEAN
    NAART_Total=MEAN VO2=MEAN) COMPARE ADJ(LSD)
  /EMMEANS=TABLES(APOE_USE*BMI4grp) WITH(Age=MEAN Education_yrs=MEAN
GENDER_USE=MEAN BDI_Total=MEAN
    NAART_Total=MEAN VO2=MEAN)
  /PRINT ETASQ DESCRIPTIVE PARAMETER HOMOGENEITY OPOWER
```

```
  /CRITERIA=ALPHA(.05)
  /DESIGN=BMI4grp APOE_USE Age Education_yrs GENDER_USE BDI_Total NAART_Total VO2
    APOE_USE*BMI4grp.
```

*******5) Left perirhinal cortex*********

```
UNIANOVA  VLeftPerirhinalCortex BY BMI4grp APOE_USE WITH Age Education_yrs
GENDER_USE BDI_Total VO2
    NAART_Total
  /CONTRAST(BMI4grp)=Repeated
  /CONTRAST(APOE_USE)=Simple(1)
  /METHOD=SSTYPE(3)
  /INTERCEPT=INCLUDE
  /PLOT=PROFILE(BMI4grp*APOE_USE BMI4grp) TYPE=LINE ERRORBAR=SE(2)
MEANREFERENCE=NO YAXIS=AUTO
  /EMMEANS=TABLES(OVERALL) WITH(Age=MEAN Education_yrs=MEAN GENDER_USE=MEAN
BDI_Total=MEAN
    NAART_Total=MEAN VO2=MEAN)
  /EMMEANS=TABLES(BMI4grp) WITH(Age=MEAN Education_yrs=MEAN GENDER_USE=MEAN
BDI_Total=MEAN
    NAART_Total=MEAN VO2=MEAN) COMPARE ADJ(LSD)
  /EMMEANS=TABLES(APOE_USE) WITH(Age=MEAN Education_yrs=MEAN GENDER_USE=MEAN
BDI_Total=MEAN
    NAART_Total=MEAN VO2=MEAN) COMPARE ADJ(LSD)
  /EMMEANS=TABLES(APOE_USE*BMI4grp) WITH(Age=MEAN Education_yrs=MEAN
GENDER_USE=MEAN BDI_Total=MEAN
    NAART_Total=MEAN VO2=MEAN)
  /PRINT ETASQ DESCRIPTIVE PARAMETER HOMOGENEITY OPOWER
  /CRITERIA=ALPHA(.05)
  /DESIGN=BMI4grp APOE_USE Age Education_yrs GENDER_USE BDI_Total NAART_Total VO2
    APOE_USE*BMI4grp.

UNIANOVA  SALeftPerirhinalCortex BY BMI4grp APOE_USE WITH Age Education_yrs
GENDER_USE BDI_Total VO2
    NAART_Total
  /CONTRAST(BMI4grp)=Repeated
  /CONTRAST(APOE_USE)=Simple(1)
  /METHOD=SSTYPE(3)
  /INTERCEPT=INCLUDE
  /PLOT=PROFILE(BMI4grp*APOE_USE BMI4grp) TYPE=LINE ERRORBAR=SE(2)
MEANREFERENCE=NO YAXIS=AUTO
  /EMMEANS=TABLES(OVERALL) WITH(Age=MEAN Education_yrs=MEAN GENDER_USE=MEAN
BDI_Total=MEAN
```

```
   NAART_Total=MEAN VO2=MEAN)
  /EMMEANS=TABLES(BMI4grp) WITH(Age=MEAN Education_yrs=MEAN GENDER_USE=MEAN
BDI_Total=MEAN
   NAART_Total=MEAN VO2=MEAN) COMPARE ADJ(LSD)
  /EMMEANS=TABLES(APOE_USE) WITH(Age=MEAN Education_yrs=MEAN GENDER_USE=MEAN
BDI_Total=MEAN
   NAART_Total=MEAN VO2=MEAN) COMPARE ADJ(LSD)
  /EMMEANS=TABLES(APOE_USE*BMI4grp) WITH(Age=MEAN Education_yrs=MEAN
GENDER_USE=MEAN BDI_Total=MEAN
   NAART_Total=MEAN VO2=MEAN)
  /PRINT ETASQ DESCRIPTIVE PARAMETER HOMOGENEITY OPOWER
  /CRITERIA=ALPHA(.05)
  /DESIGN=BMI4grp APOE_USE Age Education_yrs GENDER_USE BDI_Total NAART_Total VO2
   APOE_USE*BMI4grp.

*******6) Right perirhinal cortex*********

UNIANOVA  VRightPerirhinalCortex BY BMI4grp APOE_USE WITH Age Education_yrs
GENDER_USE BDI_Total VO2
   NAART_Total
  /CONTRAST(BMI4grp)=Repeated
  /CONTRAST(APOE_USE)=Simple(1)
  /METHOD=SSTYPE(3)
  /INTERCEPT=INCLUDE
  /PLOT=PROFILE(BMI4grp*APOE_USE BMI4grp) TYPE=LINE ERRORBAR=SE(2)
MEANREFERENCE=NO YAXIS=AUTO
  /EMMEANS=TABLES(OVERALL) WITH(Age=MEAN Education_yrs=MEAN GENDER_USE=MEAN
BDI_Total=MEAN
   NAART_Total=MEAN VO2=MEAN)
  /EMMEANS=TABLES(BMI4grp) WITH(Age=MEAN Education_yrs=MEAN GENDER_USE=MEAN
BDI_Total=MEAN
   NAART_Total=MEAN VO2=MEAN) COMPARE ADJ(LSD)
  /EMMEANS=TABLES(APOE_USE) WITH(Age=MEAN Education_yrs=MEAN GENDER_USE=MEAN
BDI_Total=MEAN
   NAART_Total=MEAN VO2=MEAN) COMPARE ADJ(LSD)
  /EMMEANS=TABLES(APOE_USE*BMI4grp) WITH(Age=MEAN Education_yrs=MEAN
GENDER_USE=MEAN BDI_Total=MEAN
   NAART_Total=MEAN VO2=MEAN)
  /PRINT ETASQ DESCRIPTIVE PARAMETER HOMOGENEITY OPOWER
  /CRITERIA=ALPHA(.05)
  /DESIGN=BMI4grp APOE_USE Age Education_yrs GENDER_USE BDI_Total NAART_Total VO2
   APOE_USE*BMI4grp.
```

```
UNIANOVA  SARightPerirhinalCortex BY BMI4grp APOE_USE WITH Age Education_yrs
GENDER_USE BDI_Total VO2
   NAART_Total
  /CONTRAST(BMI4grp)=Repeated
  /CONTRAST(APOE_USE)=Simple(1)
  /METHOD=SSTYPE(3)
  /INTERCEPT=INCLUDE
  /PLOT=PROFILE(BMI4grp*APOE_USE BMI4grp) TYPE=LINE ERRORBAR=SE(2)
MEANREFERENCE=NO YAXIS=AUTO
  /EMMEANS=TABLES(OVERALL) WITH(Age=MEAN Education_yrs=MEAN GENDER_USE=MEAN
BDI_Total=MEAN
   NAART_Total=MEAN VO2=MEAN)
  /EMMEANS=TABLES(BMI4grp) WITH(Age=MEAN Education_yrs=MEAN GENDER_USE=MEAN
BDI_Total=MEAN
   NAART_Total=MEAN VO2=MEAN) COMPARE ADJ(LSD)
  /EMMEANS=TABLES(APOE_USE) WITH(Age=MEAN Education_yrs=MEAN GENDER_USE=MEAN
BDI_Total=MEAN
   NAART_Total=MEAN VO2=MEAN) COMPARE ADJ(LSD)
  /EMMEANS=TABLES(APOE_USE*BMI4grp) WITH(Age=MEAN Education_yrs=MEAN
GENDER_USE=MEAN BDI_Total=MEAN
   NAART_Total=MEAN VO2=MEAN)
  /PRINT ETASQ DESCRIPTIVE PARAMETER HOMOGENEITY OPOWER
  /CRITERIA=ALPHA(.05)
  /DESIGN=BMI4grp APOE_USE Age Education_yrs GENDER_USE BDI_Total NAART_Total VO2
   APOE_USE*BMI4grp.

**********7) Left parahippocampal cortex************

UNIANOVA  VLeftParahippocampalCortex BY BMI4grp APOE_USE WITH Age Education_yrs
GENDER_USE BDI_Total VO2
   NAART_Total
  /CONTRAST(BMI4grp)=Repeated
  /CONTRAST(APOE_USE)=Simple(1)
  /METHOD=SSTYPE(3)
  /INTERCEPT=INCLUDE
  /PLOT=PROFILE(BMI4grp*APOE_USE BMI4grp) TYPE=LINE ERRORBAR=SE(2)
MEANREFERENCE=NO YAXIS=AUTO
  /EMMEANS=TABLES(OVERALL) WITH(Age=MEAN Education_yrs=MEAN GENDER_USE=MEAN
BDI_Total=MEAN
   NAART_Total=MEAN VO2=MEAN)
  /EMMEANS=TABLES(BMI4grp) WITH(Age=MEAN Education_yrs=MEAN GENDER_USE=MEAN
BDI_Total=MEAN
   NAART_Total=MEAN VO2=MEAN) COMPARE ADJ(LSD)
```

```
 /EMMEANS=TABLES(APOE_USE) WITH(Age=MEAN Education_yrs=MEAN GENDER_USE=MEAN
BDI_Total=MEAN
   NAART_Total=MEAN VO2=MEAN) COMPARE ADJ(LSD)
 /EMMEANS=TABLES(APOE_USE*BMI4grp) WITH(Age=MEAN Education_yrs=MEAN
GENDER_USE=MEAN BDI_Total=MEAN
   NAART_Total=MEAN VO2=MEAN)
 /PRINT ETASQ DESCRIPTIVE PARAMETER HOMOGENEITY OPOWER
 /CRITERIA=ALPHA(.05)
 /DESIGN=BMI4grp APOE_USE Age Education_yrs GENDER_USE BDI_Total NAART_Total VO2
   APOE_USE*BMI4grp.


UNIANOVA  SALeftParahippocampalCortex BY BMI4grp APOE_USE WITH Age Education_yrs
GENDER_USE BDI_Total VO2
   NAART_Total
 /CONTRAST(BMI4grp)=Repeated
 /CONTRAST(APOE_USE)=Simple(1)
 /METHOD=SSTYPE(3)
 /INTERCEPT=INCLUDE
 /PLOT=PROFILE(BMI4grp*APOE_USE BMI4grp) TYPE=LINE ERRORBAR=SE(2)
MEANREFERENCE=NO YAXIS=AUTO
 /EMMEANS=TABLES(OVERALL) WITH(Age=MEAN Education_yrs=MEAN GENDER_USE=MEAN
BDI_Total=MEAN
   NAART_Total=MEAN VO2=MEAN)
 /EMMEANS=TABLES(BMI4grp) WITH(Age=MEAN Education_yrs=MEAN GENDER_USE=MEAN
BDI_Total=MEAN
   NAART_Total=MEAN VO2=MEAN) COMPARE ADJ(LSD)
 /EMMEANS=TABLES(APOE_USE) WITH(Age=MEAN Education_yrs=MEAN GENDER_USE=MEAN
BDI_Total=MEAN
   NAART_Total=MEAN VO2=MEAN) COMPARE ADJ(LSD)
 /EMMEANS=TABLES(APOE_USE*BMI4grp) WITH(Age=MEAN Education_yrs=MEAN
GENDER_USE=MEAN BDI_Total=MEAN
   NAART_Total=MEAN VO2=MEAN)
 /PRINT ETASQ DESCRIPTIVE PARAMETER HOMOGENEITY OPOWER
 /CRITERIA=ALPHA(.05)
 /DESIGN=BMI4grp APOE_USE Age Education_yrs GENDER_USE BDI_Total NAART_Total VO2
   APOE_USE*BMI4grp.

**********8) Right parahippocampal cortex************

UNIANOVA  VRightParahippocampalCortex BY BMI4grp APOE_USE WITH Age Education_yrs
GENDER_USE BDI_Total VO2
   NAART_Total
 /CONTRAST(BMI4grp)=Repeated
```

```
  /CONTRAST(APOE_USE)=Simple(1)
  /METHOD=SSTYPE(3)
  /INTERCEPT=INCLUDE
  /PLOT=PROFILE(BMI4grp*APOE_USE BMI4grp) TYPE=LINE ERRORBAR=SE(2)
MEANREFERENCE=NO YAXIS=AUTO
  /EMMEANS=TABLES(OVERALL) WITH(Age=MEAN Education_yrs=MEAN GENDER_USE=MEAN
BDI_Total=MEAN
    NAART_Total=MEAN VO2=MEAN)
  /EMMEANS=TABLES(BMI4grp) WITH(Age=MEAN Education_yrs=MEAN GENDER_USE=MEAN
BDI_Total=MEAN
    NAART_Total=MEAN VO2=MEAN) COMPARE ADJ(LSD)
  /EMMEANS=TABLES(APOE_USE) WITH(Age=MEAN Education_yrs=MEAN GENDER_USE=MEAN
BDI_Total=MEAN
    NAART_Total=MEAN VO2=MEAN) COMPARE ADJ(LSD)
  /EMMEANS=TABLES(APOE_USE*BMI4grp) WITH(Age=MEAN Education_yrs=MEAN
GENDER_USE=MEAN BDI_Total=MEAN
    NAART_Total=MEAN VO2=MEAN)
  /PRINT ETASQ DESCRIPTIVE PARAMETER HOMOGENEITY OPOWER
  /CRITERIA=ALPHA(.05)
  /DESIGN=BMI4grp APOE_USE Age Education_yrs GENDER_USE BDI_Total NAART_Total VO2
    APOE_USE*BMI4grp.

UNIANOVA  SARightParahippocampalCortex BY BMI4grp APOE_USE WITH Age Education_yrs
GENDER_USE BDI_Total VO2
    NAART_Total
  /CONTRAST(BMI4grp)=Repeated
  /CONTRAST(APOE_USE)=Simple(1)
  /METHOD=SSTYPE(3)
  /INTERCEPT=INCLUDE
  /PLOT=PROFILE(BMI4grp*APOE_USE BMI4grp) TYPE=LINE ERRORBAR=SE(2)
MEANREFERENCE=NO YAXIS=AUTO
  /EMMEANS=TABLES(OVERALL) WITH(Age=MEAN Education_yrs=MEAN GENDER_USE=MEAN
BDI_Total=MEAN
    NAART_Total=MEAN VO2=MEAN)
  /EMMEANS=TABLES(BMI4grp) WITH(Age=MEAN Education_yrs=MEAN GENDER_USE=MEAN
BDI_Total=MEAN
    NAART_Total=MEAN VO2=MEAN) COMPARE ADJ(LSD)
  /EMMEANS=TABLES(APOE_USE) WITH(Age=MEAN Education_yrs=MEAN GENDER_USE=MEAN
BDI_Total=MEAN
    NAART_Total=MEAN VO2=MEAN) COMPARE ADJ(LSD)
  /EMMEANS=TABLES(APOE_USE*BMI4grp) WITH(Age=MEAN Education_yrs=MEAN
GENDER_USE=MEAN BDI_Total=MEAN
    NAART_Total=MEAN VO2=MEAN)
  /PRINT ETASQ DESCRIPTIVE PARAMETER HOMOGENEITY OPOWER
```

```
  /CRITERIA=ALPHA(.05)
  /DESIGN=BMI4grp APOE_USE Age Education_yrs GENDER_USE BDI_Total NAART_Total VO2
    APOE_USE*BMI4grp.
```

********9) Left DG/CA3*********

```
UNIANOVA  VLeftDGCA3 BY BMI4grp APOE_USE WITH Age Education_yrs GENDER_USE
BDI_Total VO2
    NAART_Total
  /CONTRAST(BMI4grp)=Repeated
  /CONTRAST(APOE_USE)=Simple(1)
  /METHOD=SSTYPE(3)
  /INTERCEPT=INCLUDE
  /PLOT=PROFILE(BMI4grp*APOE_USE BMI4grp) TYPE=LINE ERRORBAR=SE(2)
MEANREFERENCE=NO YAXIS=AUTO
  /EMMEANS=TABLES(OVERALL) WITH(Age=MEAN Education_yrs=MEAN GENDER_USE=MEAN
BDI_Total=MEAN
    NAART_Total=MEAN VO2=MEAN)
  /EMMEANS=TABLES(BMI4grp) WITH(Age=MEAN Education_yrs=MEAN GENDER_USE=MEAN
BDI_Total=MEAN
    NAART_Total=MEAN VO2=MEAN) COMPARE ADJ(LSD)
  /EMMEANS=TABLES(APOE_USE) WITH(Age=MEAN Education_yrs=MEAN GENDER_USE=MEAN
BDI_Total=MEAN
    NAART_Total=MEAN VO2=MEAN) COMPARE ADJ(LSD)
  /EMMEANS=TABLES(APOE_USE*BMI4grp) WITH(Age=MEAN Education_yrs=MEAN
GENDER_USE=MEAN BDI_Total=MEAN
    NAART_Total=MEAN VO2=MEAN)
  /PRINT ETASQ DESCRIPTIVE PARAMETER HOMOGENEITY OPOWER
  /CRITERIA=ALPHA(.05)
  /DESIGN=BMI4grp APOE_USE Age Education_yrs GENDER_USE BDI_Total NAART_Total VO2
    APOE_USE*BMI4grp.
```

******significant at p<.05, followed up with separate post-hoc one-way ANCOVAs fixed at APOE low and high risk******

*****low risk APOE filter*****

```
USE ALL.
COMPUTE filter_$=(APOE_USE=0).
VARIABLE LABELS filter_$ 'APOE_USE=0 (FILTER)'.
VALUE LABELS filter_$ 0 'Not Selected' 1 'Selected'.
FORMATS filter_$ (f1.0).
FILTER BY filter_$.
```

```
EXECUTE.

UNIANOVA VLeftDGCA3 BY BMI4grp WITH Age Education_yrs GENDER_USE BDI_Total
NAART_Total VO2
  /CONTRAST(BMI4grp)=Repeated
  /METHOD=SSTYPE(3)
  /INTERCEPT=INCLUDE
  /PLOT=PROFILE(BMI4grp) TYPE=LINE ERRORBAR=SE(2) MEANREFERENCE=NO YAXIS=AUTO
  /EMMEANS=TABLES(OVERALL) WITH(Age=MEAN Education_yrs=MEAN GENDER_USE=MEAN
BDI_Total=MEAN
    NAART_Total=MEAN VO2=MEAN)
  /EMMEANS=TABLES(BMI4grp) WITH(Age=MEAN Education_yrs=MEAN GENDER_USE=MEAN
BDI_Total=MEAN
    NAART_Total=MEAN VO2=MEAN) COMPARE ADJ(LSD)
  /PRINT ETASQ DESCRIPTIVE PARAMETER HOMOGENEITY OPOWER
  /CRITERIA=ALPHA(.05)
  /DESIGN=BMI4grp Age Education_yrs GENDER_USE BDI_Total NAART_Total VO2.

*****high risk APOE filter*****

USE ALL.
COMPUTE filter_$=(APOE_USE=1).
VARIABLE LABELS filter_$ 'APOE_USE=1 (FILTER)'.
VALUE LABELS filter_$ 0 'Not Selected' 1 'Selected'.
FORMATS filter_$ (f1.0).
FILTER BY filter_$.
EXECUTE.

UNIANOVA VLeftDGCA3 BY BMI4grp WITH Age Education_yrs GENDER_USE BDI_Total
NAART_Total VO2
  /CONTRAST(BMI4grp)=Repeated
  /METHOD=SSTYPE(3)
  /INTERCEPT=INCLUDE
  /PLOT=PROFILE(BMI4grp) TYPE=LINE ERRORBAR=SE(2) MEANREFERENCE=NO YAXIS=AUTO
  /EMMEANS=TABLES(OVERALL) WITH(Age=MEAN Education_yrs=MEAN GENDER_USE=MEAN
BDI_Total=MEAN
    NAART_Total=MEAN VO2=MEAN)
  /EMMEANS=TABLES(BMI4grp) WITH(Age=MEAN Education_yrs=MEAN GENDER_USE=MEAN
BDI_Total=MEAN
    NAART_Total=MEAN VO2=MEAN) COMPARE ADJ(LSD)
  /PRINT ETASQ DESCRIPTIVE PARAMETER HOMOGENEITY OPOWER
  /CRITERIA=ALPHA(.05)
  /DESIGN=BMI4grp Age Education_yrs GENDER_USE BDI_Total NAART_Total VO2.
```

```
FILTER OFF.
USE ALL.
EXECUTE.

*****back to left DG/CA3 analyses- surface area*****

UNIANOVA  SALeftDGCA3  BY BMI4grp APOE_USE WITH Age Education_yrs GENDER_USE
BDI_Total VO2
    NAART_Total
  /CONTRAST(BMI4grp)=Repeated
  /CONTRAST(APOE_USE)=Simple(1)
  /METHOD=SSTYPE(3)
  /INTERCEPT=INCLUDE
  /PLOT=PROFILE(BMI4grp*APOE_USE BMI4grp) TYPE=LINE ERRORBAR=SE(2)
MEANREFERENCE=NO YAXIS=AUTO
  /EMMEANS=TABLES(OVERALL) WITH(Age=MEAN Education_yrs=MEAN GENDER_USE=MEAN
BDI_Total=MEAN
    NAART_Total=MEAN VO2=MEAN)
  /EMMEANS=TABLES(BMI4grp) WITH(Age=MEAN Education_yrs=MEAN GENDER_USE=MEAN
BDI_Total=MEAN
    NAART_Total=MEAN VO2=MEAN) COMPARE ADJ(LSD)
  /EMMEANS=TABLES(APOE_USE) WITH(Age=MEAN Education_yrs=MEAN GENDER_USE=MEAN
BDI_Total=MEAN
    NAART_Total=MEAN VO2=MEAN) COMPARE ADJ(LSD)
  /EMMEANS=TABLES(APOE_USE*BMI4grp) WITH(Age=MEAN Education_yrs=MEAN
GENDER_USE=MEAN BDI_Total=MEAN
    NAART_Total=MEAN VO2=MEAN)
  /PRINT ETASQ DESCRIPTIVE PARAMETER HOMOGENEITY OPOWER
  /CRITERIA=ALPHA(.05)
  /DESIGN=BMI4grp APOE_USE Age Education_yrs GENDER_USE BDI_Total NAART_Total VO2
    APOE_USE*BMI4grp.

******trending p = .06, followed up with separate post-hoc one-way ANCOVAs fixed at APOE
low and high risk******

*****low risk APOE filter*****

USE ALL.
COMPUTE filter_$=(APOE_USE=0).
VARIABLE LABELS filter_$ 'APOE_USE=0 (FILTER)'.
VALUE LABELS filter_$ 0 'Not Selected' 1 'Selected'.
FORMATS filter_$ (f1.0).
FILTER BY filter_$.
EXECUTE.
```

```
UNIANOVA  SALeftDGCA3 BY BMI4grp WITH Age Education_yrs GENDER_USE BDI_Total
NAART_Total VO2
  /CONTRAST(BMI4grp)=Repeated
  /METHOD=SSTYPE(3)
  /INTERCEPT=INCLUDE
  /PLOT=PROFILE(BMI4grp) TYPE=LINE ERRORBAR=SE(2) MEANREFERENCE=NO YAXIS=AUTO
  /EMMEANS=TABLES(OVERALL) WITH(Age=MEAN Education_yrs=MEAN GENDER_USE=MEAN
BDI_Total=MEAN
    NAART_Total=MEAN VO2=MEAN)
  /EMMEANS=TABLES(BMI4grp) WITH(Age=MEAN Education_yrs=MEAN GENDER_USE=MEAN
BDI_Total=MEAN
    NAART_Total=MEAN VO2=MEAN) COMPARE ADJ(LSD)
  /PRINT ETASQ DESCRIPTIVE PARAMETER HOMOGENEITY OPOWER
  /CRITERIA=ALPHA(.05)
  /DESIGN=BMI4grp Age Education_yrs GENDER_USE BDI_Total NAART_Total VO2.

*****high risk APOE filter*****

USE ALL.
COMPUTE filter_$=(APOE_USE=1).
VARIABLE LABELS filter_$ 'APOE_USE=1 (FILTER)'.
VALUE LABELS filter_$ 0 'Not Selected' 1 'Selected'.
FORMATS filter_$ (f1.0).
FILTER BY filter_$.
EXECUTE.

UNIANOVA SALeftDGCA3 BY BMI4grp WITH Age Education_yrs GENDER_USE BDI_Total
NAART_Total VO2
  /CONTRAST(BMI4grp)=Repeated
  /METHOD=SSTYPE(3)
  /INTERCEPT=INCLUDE
  /PLOT=PROFILE(BMI4grp) TYPE=LINE ERRORBAR=SE(2) MEANREFERENCE=NO YAXIS=AUTO
  /EMMEANS=TABLES(OVERALL) WITH(Age=MEAN Education_yrs=MEAN GENDER_USE=MEAN
BDI_Total=MEAN
    NAART_Total=MEAN VO2=MEAN)
  /EMMEANS=TABLES(BMI4grp) WITH(Age=MEAN Education_yrs=MEAN GENDER_USE=MEAN
BDI_Total=MEAN
    NAART_Total=MEAN VO2=MEAN) COMPARE ADJ(LSD)
  /PRINT ETASQ DESCRIPTIVE PARAMETER HOMOGENEITY OPOWER
  /CRITERIA=ALPHA(.05)
  /DESIGN=BMI4grp Age Education_yrs GENDER_USE BDI_Total NAART_Total VO2.

FILTER OFF.
```

```
USE ALL.
EXECUTE.

********10) Right DG/CA3*********

UNIANOVA  VRightDGCA3 BY BMI4grp APOE_USE WITH Age Education_yrs GENDER_USE
BDI_Total VO2
   NAART_Total
  /CONTRAST(BMI4grp)=Repeated
  /CONTRAST(APOE_USE)=Simple(1)
  /METHOD=SSTYPE(3)
  /INTERCEPT=INCLUDE
  /PLOT=PROFILE(BMI4grp*APOE_USE BMI4grp) TYPE=LINE ERRORBAR=SE(2)
MEANREFERENCE=NO YAXIS=AUTO
  /EMMEANS=TABLES(OVERALL) WITH(Age=MEAN Education_yrs=MEAN GENDER_USE=MEAN
BDI_Total=MEAN
   NAART_Total=MEAN VO2=MEAN)
  /EMMEANS=TABLES(BMI4grp) WITH(Age=MEAN Education_yrs=MEAN GENDER_USE=MEAN
BDI_Total=MEAN
   NAART_Total=MEAN VO2=MEAN) COMPARE ADJ(LSD)
  /EMMEANS=TABLES(APOE_USE) WITH(Age=MEAN Education_yrs=MEAN GENDER_USE=MEAN
BDI_Total=MEAN
   NAART_Total=MEAN VO2=MEAN) COMPARE ADJ(LSD)
  /EMMEANS=TABLES(APOE_USE*BMI4grp) WITH(Age=MEAN Education_yrs=MEAN
GENDER_USE=MEAN BDI_Total=MEAN
   NAART_Total=MEAN VO2=MEAN)
  /PRINT ETASQ DESCRIPTIVE PARAMETER HOMOGENEITY OPOWER
  /CRITERIA=ALPHA(.05)
  /DESIGN=BMI4grp APOE_USE Age Education_yrs GENDER_USE BDI_Total NAART_Total VO2
   APOE_USE*BMI4grp.

UNIANOVA  SARightDGCA3 BY BMI4grp APOE_USE WITH Age Education_yrs GENDER_USE
BDI_Total VO2
   NAART_Total
  /CONTRAST(BMI4grp)=Repeated
  /CONTRAST(APOE_USE)=Simple(1)
  /METHOD=SSTYPE(3)
  /INTERCEPT=INCLUDE
  /PLOT=PROFILE(BMI4grp*APOE_USE BMI4grp) TYPE=LINE ERRORBAR=SE(2)
MEANREFERENCE=NO YAXIS=AUTO
  /EMMEANS=TABLES(OVERALL) WITH(Age=MEAN Education_yrs=MEAN GENDER_USE=MEAN
BDI_Total=MEAN
   NAART_Total=MEAN VO2=MEAN)
```

```
  /EMMEANS=TABLES(BMI4grp) WITH(Age=MEAN Education_yrs=MEAN GENDER_USE=MEAN
BDI_Total=MEAN
    NAART_Total=MEAN VO2=MEAN) COMPARE ADJ(LSD)
  /EMMEANS=TABLES(APOE_USE) WITH(Age=MEAN Education_yrs=MEAN GENDER_USE=MEAN
BDI_Total=MEAN
    NAART_Total=MEAN VO2=MEAN) COMPARE ADJ(LSD)
  /EMMEANS=TABLES(APOE_USE*BMI4grp) WITH(Age=MEAN Education_yrs=MEAN
GENDER_USE=MEAN BDI_Total=MEAN
    NAART_Total=MEAN VO2=MEAN)
  /PRINT ETASQ DESCRIPTIVE PARAMETER HOMOGENEITY OPOWER
  /CRITERIA=ALPHA(.05)
  /DESIGN=BMI4grp APOE_USE Age Education_yrs GENDER_USE BDI_Total NAART_Total VO2
    APOE_USE*BMI4grp.
```

*******11) Left CA1*********

```
UNIANOVA   VLeftCA1 BY BMI4grp APOE_USE WITH Age Education_yrs GENDER_USE BDI_Total
VO2
    NAART_Total
  /CONTRAST(BMI4grp)=Repeated
  /CONTRAST(APOE_USE)=Simple(1)
  /METHOD=SSTYPE(3)
  /INTERCEPT=INCLUDE
  /PLOT=PROFILE(BMI4grp*APOE_USE BMI4grp) TYPE=LINE ERRORBAR=SE(2)
MEANREFERENCE=NO YAXIS=AUTO
  /EMMEANS=TABLES(OVERALL) WITH(Age=MEAN Education_yrs=MEAN GENDER_USE=MEAN
BDI_Total=MEAN
    NAART_Total=MEAN VO2=MEAN)
  /EMMEANS=TABLES(BMI4grp) WITH(Age=MEAN Education_yrs=MEAN GENDER_USE=MEAN
BDI_Total=MEAN
    NAART_Total=MEAN VO2=MEAN) COMPARE ADJ(LSD)
  /EMMEANS=TABLES(APOE_USE) WITH(Age=MEAN Education_yrs=MEAN GENDER_USE=MEAN
BDI_Total=MEAN
    NAART_Total=MEAN VO2=MEAN) COMPARE ADJ(LSD)
  /EMMEANS=TABLES(APOE_USE*BMI4grp) WITH(Age=MEAN Education_yrs=MEAN
GENDER_USE=MEAN BDI_Total=MEAN
    NAART_Total=MEAN VO2=MEAN)
  /PRINT ETASQ DESCRIPTIVE PARAMETER HOMOGENEITY OPOWER
  /CRITERIA=ALPHA(.05)
  /DESIGN=BMI4grp APOE_USE Age Education_yrs GENDER_USE BDI_Total NAART_Total VO2
    APOE_USE*BMI4grp.
```

******significant at p<.05, followed up with separate post-hoc one-way ANCOVAs fixed at APOE low and high risk******

```
*****low risk APOE filter*****

USE ALL.
COMPUTE filter_$=(APOE_USE=0).
VARIABLE LABELS filter_$ 'APOE_USE=0 (FILTER)'.
VALUE LABELS filter_$ 0 'Not Selected' 1 'Selected'.
FORMATS filter_$ (f1.0).
FILTER BY filter_$.
EXECUTE.

UNIANOVA   VLeftCA1 BY BMI4grp WITH Age Education_yrs GENDER_USE BDI_Total
NAART_Total VO2
  /CONTRAST(BMI4grp)=Repeated
  /METHOD=SSTYPE(3)
  /INTERCEPT=INCLUDE
  /PLOT=PROFILE(BMI4grp) TYPE=LINE ERRORBAR=SE(2) MEANREFERENCE=NO YAXIS=AUTO
  /EMMEANS=TABLES(OVERALL) WITH(Age=MEAN Education_yrs=MEAN GENDER_USE=MEAN
BDI_Total=MEAN
    NAART_Total=MEAN VO2=MEAN)
  /EMMEANS=TABLES(BMI4grp) WITH(Age=MEAN Education_yrs=MEAN GENDER_USE=MEAN
BDI_Total=MEAN
    NAART_Total=MEAN VO2=MEAN) COMPARE ADJ(LSD)
  /PRINT ETASQ DESCRIPTIVE PARAMETER HOMOGENEITY OPOWER
  /CRITERIA=ALPHA(.05)
  /DESIGN=BMI4grp Age Education_yrs GENDER_USE BDI_Total NAART_Total VO2.

*****high risk APOE filter*****

USE ALL.
COMPUTE filter_$=(APOE_USE=1).
VARIABLE LABELS filter_$ 'APOE_USE=1 (FILTER)'.
VALUE LABELS filter_$ 0 'Not Selected' 1 'Selected'.
FORMATS filter_$ (f1.0).
FILTER BY filter_$.
EXECUTE.

UNIANOVA VLeftCA1 BY BMI4grp WITH Age Education_yrs GENDER_USE BDI_Total NAART_Total
VO2
  /CONTRAST(BMI4grp)=Repeated
  /METHOD=SSTYPE(3)
  /INTERCEPT=INCLUDE
  /PLOT=PROFILE(BMI4grp) TYPE=LINE ERRORBAR=SE(2) MEANREFERENCE=NO YAXIS=AUTO
```

```
  /EMMEANS=TABLES(OVERALL) WITH(Age=MEAN Education_yrs=MEAN GENDER_USE=MEAN
BDI_Total=MEAN
    NAART_Total=MEAN VO2=MEAN)
  /EMMEANS=TABLES(BMI4grp) WITH(Age=MEAN Education_yrs=MEAN GENDER_USE=MEAN
BDI_Total=MEAN
    NAART_Total=MEAN VO2=MEAN) COMPARE ADJ(LSD)
  /PRINT ETASQ DESCRIPTIVE PARAMETER HOMOGENEITY OPOWER
  /CRITERIA=ALPHA(.05)
  /DESIGN=BMI4grp Age Education_yrs GENDER_USE BDI_Total NAART_Total VO2.

FILTER OFF.
USE ALL.
EXECUTE.

****left CA1 analyses continued-  surface area ******

UNIANOVA   SALeftCA1 BY BMI4grp APOE_USE WITH Age Education_yrs GENDER_USE BDI_Total
VO2
    NAART_Total
  /CONTRAST(BMI4grp)=Repeated
  /CONTRAST(APOE_USE)=Simple(1)
  /METHOD=SSTYPE(3)
  /INTERCEPT=INCLUDE
  /PLOT=PROFILE(BMI4grp*APOE_USE BMI4grp) TYPE=LINE ERRORBAR=SE(2)
MEANREFERENCE=NO YAXIS=AUTO
  /EMMEANS=TABLES(OVERALL) WITH(Age=MEAN Education_yrs=MEAN GENDER_USE=MEAN
BDI_Total=MEAN
    NAART_Total=MEAN VO2=MEAN)
  /EMMEANS=TABLES(BMI4grp) WITH(Age=MEAN Education_yrs=MEAN GENDER_USE=MEAN
BDI_Total=MEAN
    NAART_Total=MEAN VO2=MEAN) COMPARE ADJ(LSD)
  /EMMEANS=TABLES(APOE_USE) WITH(Age=MEAN Education_yrs=MEAN GENDER_USE=MEAN
BDI_Total=MEAN
    NAART_Total=MEAN VO2=MEAN) COMPARE ADJ(LSD)
  /EMMEANS=TABLES(APOE_USE*BMI4grp) WITH(Age=MEAN Education_yrs=MEAN
GENDER_USE=MEAN BDI_Total=MEAN
    NAART_Total=MEAN VO2=MEAN)
  /PRINT ETASQ DESCRIPTIVE PARAMETER HOMOGENEITY OPOWER
  /CRITERIA=ALPHA(.05)
  /DESIGN=BMI4grp APOE_USE Age Education_yrs GENDER_USE BDI_Total NAART_Total VO2
    APOE_USE*BMI4grp.

******significant at p<.05, followed up with separate post-hoc one-way ANCOVAs fixed at APOE
low and high risk******
```

```
*****low risk APOE filter*****

USE ALL.
COMPUTE filter_$=(APOE_USE=0).
VARIABLE LABELS filter_$ 'APOE_USE=0 (FILTER)'.
VALUE LABELS filter_$ 0 'Not Selected' 1 'Selected'.
FORMATS filter_$ (f1.0).
FILTER BY filter_$.
EXECUTE.

UNIANOVA  SALeftCA1 BY BMI4grp WITH Age Education_yrs GENDER_USE BDI_Total
NAART_Total VO2
  /CONTRAST(BMI4grp)=Repeated
  /METHOD=SSTYPE(3)
  /INTERCEPT=INCLUDE
  /PLOT=PROFILE(BMI4grp) TYPE=LINE ERRORBAR=SE(2) MEANREFERENCE=NO YAXIS=AUTO
  /EMMEANS=TABLES(OVERALL) WITH(Age=MEAN Education_yrs=MEAN GENDER_USE=MEAN
BDI_Total=MEAN
    NAART_Total=MEAN VO2=MEAN)
  /EMMEANS=TABLES(BMI4grp) WITH(Age=MEAN Education_yrs=MEAN GENDER_USE=MEAN
BDI_Total=MEAN
    NAART_Total=MEAN VO2=MEAN) COMPARE ADJ(LSD)
  /PRINT ETASQ DESCRIPTIVE PARAMETER HOMOGENEITY OPOWER
  /CRITERIA=ALPHA(.05)
  /DESIGN=BMI4grp Age Education_yrs GENDER_USE BDI_Total NAART_Total VO2.

*****high risk APOE filter*****

USE ALL.
COMPUTE filter_$=(APOE_USE=1).
VARIABLE LABELS filter_$ 'APOE_USE=1 (FILTER)'.
VALUE LABELS filter_$ 0 'Not Selected' 1 'Selected'.
FORMATS filter_$ (f1.0).
FILTER BY filter_$.
EXECUTE.

UNIANOVA SALeftCA1 BY BMI4grp WITH Age Education_yrs GENDER_USE BDI_Total
NAART_Total VO2
  /CONTRAST(BMI4grp)=Repeated
  /METHOD=SSTYPE(3)
  /INTERCEPT=INCLUDE
  /PLOT=PROFILE(BMI4grp) TYPE=LINE ERRORBAR=SE(2) MEANREFERENCE=NO YAXIS=AUTO
```

```
  /EMMEANS=TABLES(OVERALL) WITH(Age=MEAN Education_yrs=MEAN GENDER_USE=MEAN
BDI_Total=MEAN
    NAART_Total=MEAN VO2=MEAN)
  /EMMEANS=TABLES(BMI4grp) WITH(Age=MEAN Education_yrs=MEAN GENDER_USE=MEAN
BDI_Total=MEAN
    NAART_Total=MEAN VO2=MEAN) COMPARE ADJ(LSD)
  /PRINT ETASQ DESCRIPTIVE PARAMETER HOMOGENEITY OPOWER
  /CRITERIA=ALPHA(.05)
  /DESIGN=BMI4grp Age Education_yrs GENDER_USE BDI_Total NAART_Total VO2.

FILTER OFF.
USE ALL.
EXECUTE.

********12) Right CA1*********

UNIANOVA  VRightCA1 BY BMI4grp APOE_USE WITH Age Education_yrs GENDER_USE BDI_Total
VO2
    NAART_Total
  /CONTRAST(BMI4grp)=Repeated
  /CONTRAST(APOE_USE)=Simple(1)
  /METHOD=SSTYPE(3)
  /INTERCEPT=INCLUDE
  /PLOT=PROFILE(BMI4grp*APOE_USE BMI4grp) TYPE=LINE ERRORBAR=SE(2)
MEANREFERENCE=NO YAXIS=AUTO
  /EMMEANS=TABLES(OVERALL) WITH(Age=MEAN Education_yrs=MEAN GENDER_USE=MEAN
BDI_Total=MEAN
    NAART_Total=MEAN VO2=MEAN)
  /EMMEANS=TABLES(BMI4grp) WITH(Age=MEAN Education_yrs=MEAN GENDER_USE=MEAN
BDI_Total=MEAN
    NAART_Total=MEAN VO2=MEAN) COMPARE ADJ(LSD)
  /EMMEANS=TABLES(APOE_USE) WITH(Age=MEAN Education_yrs=MEAN GENDER_USE=MEAN
BDI_Total=MEAN
    NAART_Total=MEAN VO2=MEAN) COMPARE ADJ(LSD)
  /EMMEANS=TABLES(APOE_USE*BMI4grp) WITH(Age=MEAN Education_yrs=MEAN
GENDER_USE=MEAN BDI_Total=MEAN
    NAART_Total=MEAN VO2=MEAN)
  /PRINT ETASQ DESCRIPTIVE PARAMETER HOMOGENEITY OPOWER
  /CRITERIA=ALPHA(.05)
  /DESIGN=BMI4grp APOE_USE Age Education_yrs GENDER_USE BDI_Total NAART_Total VO2
    APOE_USE*BMI4grp.
```

```
UNIANOVA  SARightCA1 BY BMI4grp APOE_USE WITH Age Education_yrs GENDER_USE
BDI_Total VO2
    NAART_Total
  /CONTRAST(BMI4grp)=Repeated
  /CONTRAST(APOE_USE)=Simple(1)
  /METHOD=SSTYPE(3)
  /INTERCEPT=INCLUDE
  /PLOT=PROFILE(BMI4grp*APOE_USE BMI4grp) TYPE=LINE ERRORBAR=SE(2)
MEANREFERENCE=NO YAXIS=AUTO
  /EMMEANS=TABLES(OVERALL) WITH(Age=MEAN Education_yrs=MEAN GENDER_USE=MEAN
BDI_Total=MEAN
    NAART_Total=MEAN VO2=MEAN)
  /EMMEANS=TABLES(BMI4grp) WITH(Age=MEAN Education_yrs=MEAN GENDER_USE=MEAN
BDI_Total=MEAN
    NAART_Total=MEAN VO2=MEAN) COMPARE ADJ(LSD)
  /EMMEANS=TABLES(APOE_USE) WITH(Age=MEAN Education_yrs=MEAN GENDER_USE=MEAN
BDI_Total=MEAN
    NAART_Total=MEAN VO2=MEAN) COMPARE ADJ(LSD)
  /EMMEANS=TABLES(APOE_USE*BMI4grp) WITH(Age=MEAN Education_yrs=MEAN
GENDER_USE=MEAN BDI_Total=MEAN
    NAART_Total=MEAN VO2=MEAN)
  /PRINT ETASQ DESCRIPTIVE PARAMETER HOMOGENEITY OPOWER
  /CRITERIA=ALPHA(.05)
  /DESIGN=BMI4grp APOE_USE Age Education_yrs GENDER_USE BDI_Total NAART_Total VO2
    APOE_USE*BMI4grp.
```

********13) Left subiculum*********

```
UNIANOVA  VLeftSubiculum BY BMI4grp APOE_USE WITH Age Education_yrs GENDER_USE
BDI_Total VO2
    NAART_Total
  /CONTRAST(BMI4grp)=Repeated
  /CONTRAST(APOE_USE)=Simple(1)
  /METHOD=SSTYPE(3)
  /INTERCEPT=INCLUDE
  /PLOT=PROFILE(BMI4grp*APOE_USE BMI4grp) TYPE=LINE ERRORBAR=SE(2)
MEANREFERENCE=NO YAXIS=AUTO
  /EMMEANS=TABLES(OVERALL) WITH(Age=MEAN Education_yrs=MEAN GENDER_USE=MEAN
BDI_Total=MEAN
    NAART_Total=MEAN VO2=MEAN)
  /EMMEANS=TABLES(BMI4grp) WITH(Age=MEAN Education_yrs=MEAN GENDER_USE=MEAN
BDI_Total=MEAN
    NAART_Total=MEAN VO2=MEAN) COMPARE ADJ(LSD)
```

```
  /EMMEANS=TABLES(APOE_USE) WITH(Age=MEAN Education_yrs=MEAN GENDER_USE=MEAN
BDI_Total=MEAN
    NAART_Total=MEAN VO2=MEAN) COMPARE ADJ(LSD)
  /EMMEANS=TABLES(APOE_USE*BMI4grp) WITH(Age=MEAN Education_yrs=MEAN
GENDER_USE=MEAN BDI_Total=MEAN
    NAART_Total=MEAN VO2=MEAN)
  /PRINT ETASQ DESCRIPTIVE PARAMETER HOMOGENEITY OPOWER
  /CRITERIA=ALPHA(.05)
  /DESIGN=BMI4grp APOE_USE Age Education_yrs GENDER_USE BDI_Total NAART_Total VO2
    APOE_USE*BMI4grp.

UNIANOVA  SALeftSubiculum BY BMI4grp APOE_USE WITH Age Education_yrs GENDER_USE
BDI_Total VO2
    NAART_Total
  /CONTRAST(BMI4grp)=Repeated
  /CONTRAST(APOE_USE)=Simple(1)
  /METHOD=SSTYPE(3)
  /INTERCEPT=INCLUDE
  /PLOT=PROFILE(BMI4grp*APOE_USE BMI4grp) TYPE=LINE ERRORBAR=SE(2)
MEANREFERENCE=NO YAXIS=AUTO
  /EMMEANS=TABLES(OVERALL) WITH(Age=MEAN Education_yrs=MEAN GENDER_USE=MEAN
BDI_Total=MEAN
    NAART_Total=MEAN VO2=MEAN)
  /EMMEANS=TABLES(BMI4grp) WITH(Age=MEAN Education_yrs=MEAN GENDER_USE=MEAN
BDI_Total=MEAN
    NAART_Total=MEAN VO2=MEAN) COMPARE ADJ(LSD)
  /EMMEANS=TABLES(APOE_USE) WITH(Age=MEAN Education_yrs=MEAN GENDER_USE=MEAN
BDI_Total=MEAN
    NAART_Total=MEAN VO2=MEAN) COMPARE ADJ(LSD)
  /EMMEANS=TABLES(APOE_USE*BMI4grp) WITH(Age=MEAN Education_yrs=MEAN
GENDER_USE=MEAN BDI_Total=MEAN
    NAART_Total=MEAN VO2=MEAN)
  /PRINT ETASQ DESCRIPTIVE PARAMETER HOMOGENEITY OPOWER
  /CRITERIA=ALPHA(.05)
  /DESIGN=BMI4grp APOE_USE Age Education_yrs GENDER_USE BDI_Total NAART_Total VO2
    APOE_USE*BMI4grp.


********14) Right subiculum*********

UNIANOVA  VRightSubiculum BY BMI4grp APOE_USE WITH Age Education_yrs GENDER_USE
BDI_Total VO2
    NAART_Total
  /CONTRAST(BMI4grp)=Repeated
```

```
  /CONTRAST(APOE_USE)=Simple(1)
  /METHOD=SSTYPE(3)
  /INTERCEPT=INCLUDE
  /PLOT=PROFILE(BMI4grp*APOE_USE BMI4grp) TYPE=LINE ERRORBAR=SE(2)
MEANREFERENCE=NO YAXIS=AUTO
  /EMMEANS=TABLES(OVERALL) WITH(Age=MEAN Education_yrs=MEAN GENDER_USE=MEAN
BDI_Total=MEAN
    NAART_Total=MEAN VO2=MEAN)
  /EMMEANS=TABLES(BMI4grp) WITH(Age=MEAN Education_yrs=MEAN GENDER_USE=MEAN
BDI_Total=MEAN
    NAART_Total=MEAN VO2=MEAN) COMPARE ADJ(LSD)
  /EMMEANS=TABLES(APOE_USE) WITH(Age=MEAN Education_yrs=MEAN GENDER_USE=MEAN
BDI_Total=MEAN
    NAART_Total=MEAN VO2=MEAN) COMPARE ADJ(LSD)
  /EMMEANS=TABLES(APOE_USE*BMI4grp) WITH(Age=MEAN Education_yrs=MEAN
GENDER_USE=MEAN BDI_Total=MEAN
    NAART_Total=MEAN VO2=MEAN)
  /PRINT ETASQ DESCRIPTIVE PARAMETER HOMOGENEITY OPOWER
  /CRITERIA=ALPHA(.05)
  /DESIGN=BMI4grp APOE_USE Age Education_yrs GENDER_USE BDI_Total NAART_Total VO2
    APOE_USE*BMI4grp.

UNIANOVA   SARightSubiculum BY BMI4grp APOE_USE WITH Age Education_yrs GENDER_USE
BDI_Total VO2
    NAART_Total
  /CONTRAST(BMI4grp)=Repeated
  /CONTRAST(APOE_USE)=Simple(1)
  /METHOD=SSTYPE(3)
  /INTERCEPT=INCLUDE
  /PLOT=PROFILE(BMI4grp*APOE_USE BMI4grp) TYPE=LINE ERRORBAR=SE(2)
MEANREFERENCE=NO YAXIS=AUTO
  /EMMEANS=TABLES(OVERALL) WITH(Age=MEAN Education_yrs=MEAN GENDER_USE=MEAN
BDI_Total=MEAN
    NAART_Total=MEAN VO2=MEAN)
  /EMMEANS=TABLES(BMI4grp) WITH(Age=MEAN Education_yrs=MEAN GENDER_USE=MEAN
BDI_Total=MEAN
    NAART_Total=MEAN VO2=MEAN) COMPARE ADJ(LSD)
  /EMMEANS=TABLES(APOE_USE) WITH(Age=MEAN Education_yrs=MEAN GENDER_USE=MEAN
BDI_Total=MEAN
    NAART_Total=MEAN VO2=MEAN) COMPARE ADJ(LSD)
  /EMMEANS=TABLES(APOE_USE*BMI4grp) WITH(Age=MEAN Education_yrs=MEAN
GENDER_USE=MEAN BDI_Total=MEAN
    NAART_Total=MEAN VO2=MEAN)
  /PRINT ETASQ DESCRIPTIVE PARAMETER HOMOGENEITY OPOWER
```

```
 /CRITERIA=ALPHA(.05)
 /DESIGN=BMI4grp APOE_USE Age Education_yrs GENDER_USE BDI_Total NAART_Total VO2
   APOE_USE*BMI4grp.

**************************************************************************************
**********************************
Differences in VO2max between BMI groups
**************************************************************************************
***********

UNIANOVA   VO2 BY BMI4grp WITH Age Education_yrs GENDER_USE
 /CONTRAST(BMI4grp)=Repeated
 /METHOD=SSTYPE(3)
 /INTERCEPT=INCLUDE
 /PLOT=PROFILE(BMI4grp) TYPE=LINE ERRORBAR=SE(2) MEANREFERENCE=NO YAXIS=AUTO
 /EMMEANS=TABLES(OVERALL) WITH(Age=MEAN Education_yrs=MEAN GENDER_USE=MEAN)
 /EMMEANS=TABLES(BMI4grp) WITH(Age=MEAN Education_yrs=MEAN GENDER_USE=MEAN)
COMPARE ADJ(LSD)
 /PRINT ETASQ DESCRIPTIVE PARAMETER HOMOGENEITY OPOWER
 /CRITERIA=ALPHA(.05)
 /DESIGN=BMI4grp Age Education_yrs GENDER_USE.


*********Process model 1 syntax for moderation******
******testing for BMIxVO2 interaction on probe errors (not significant)****

**************************************************************************
Model  : 1
   Y  : ProbeErr
   X  : BMI4grp
   W  : VO2

Covariates:
 Age     Edu     GENDER1  APOE_USE BDI     NAART    TrainERR

Sample
Size:  67

Coding of categorical X variable for analysis:
 BMI4grp      X1      X2      X3
    .000    .000    .000    .000
   1.000   1.000    .000    .000
   2.000    .000   1.000    .000
   3.000    .000    .000   1.000
```

****

OUTCOME VARIABLE:
ProbeErr

Model Summary

R	R-sq	MSE	F	df1	df2	p
.8264	.6830	97.9712	8.0028	14.0000	52.0000	.0000

Model

	coeff	se	t	p	LLCI	ULCI
constant	-20.9267	31.0248	-.6745	.5030	-83.1829	41.3295
X1	-10.7036	24.0556	-.4450	.6582	-58.9751	37.5680
X2	17.7027	25.7755	.6868	.4953	-34.0201	69.4255
X3	-.7672	28.5107	-.0269	.9786	-57.9786	56.4442
VO2	.3796	1.2629	.3006	.7649	-2.1546	2.9139
Int_1	.1043	1.4882	.0701	.9444	-2.8819	3.0905
Int_2	-1.9070	1.6627	-1.1469	.2567	-5.2433	1.4294
Int_3	-.1271	1.8508	-.0687	.9455	-3.8409	3.5868
Age	.3573	.2132	1.6760	.0997	-.0705	.7851
Edu	-1.0182	.6965	-1.4619	.1498	-2.4159	.3794
GENDER1	2.5545	3.6200	.7057	.4836	-4.7096	9.8185
APOE_USE	3.4974	2.6506	1.3195	.1928	-1.8215	8.8163
BDI	-.2225	.2283	-.9745	.3343	-.6807	.2357
NAART	.1438	.1326	1.0840	.2834	-.1224	.4099
TrainERR	.6822	.0970	7.0364	.0000	.4877	.8768

Product terms key:

Int_1	:	X1	x	VO2
Int_2	:	X2	x	VO2
Int_3	:	X3	x	VO2

DATA LIST FREE/
BMI4grp VO2 ProbeErr .
BEGIN DATA.

BMI4grp	VO2	ProbeErr
.0000	12.4420	14.9924
1.0000	12.4420	5.5867
2.0000	12.4420	8.9686
3.0000	12.4420	12.6443
.0000	14.8775	15.9169
1.0000	14.8775	6.7653
2.0000	14.8775	5.2489
3.0000	14.8775	13.2594
.0000	17.5542	16.9330

```
    1.0000    17.5542     8.0607
    2.0000    17.5542     1.1607
    3.0000    17.5542    13.9354
END DATA.
GRAPH/SCATTERPLOT=
 VO2      WITH     ProbeErr BY      BMI4grp  .



*********testing BMIxVO2 interaction on VRaLEC (not significant)*****

DATA LIST FREE/
   BMI4grp    VO2       VRaLEC     .
BEGIN DATA.
     .0000    12.4420   439.5487
    1.0000    12.4420   467.9244
    2.0000    12.4420   469.2298
    3.0000    12.4420   387.0595
     .0000    14.8775   459.2160
    1.0000    14.8775   471.3236
    2.0000    14.8775   487.4917
    3.0000    14.8775   426.5407
     .0000    17.5542   480.8319
    1.0000    17.5542   475.0597
    2.0000    17.5542   507.5629
    3.0000    17.5542   469.9339
END DATA.
GRAPH/SCATTERPLOT=
 VO2      WITH     VRaLEC   BY      BMI4grp  .


************Updated Analyses********* for revise and resubmit*******

******Cognitive outcomes 11 May 2023*(********

FREQUENCIES VARIABLES=ICV
  /ORDER=ANALYSIS.

*************************COGNITIVE
OUTCOMES*************************************
    *******May 18, 2023*******

******1) Choose Probe Errors******
```

UNIANOVA ChooseProbenErr BY BMI4grp APOE_USE WITH Age Education_yrs GENDER_USE BDI_Total
   NAART_Total ChooseTrainingnErr VO2
  /CONTRAST(BMI4grp)=Repeated
  /CONTRAST(APOE_USE)=Simple(1)
  /METHOD=SSTYPE(3)
  /INTERCEPT=INCLUDE
  /PLOT=PROFILE(BMI4grp*APOE_USE BMI4grp) TYPE=LINE ERRORBAR=SE(2) MEANREFERENCE=NO YAXIS=AUTO
  /EMMEANS=TABLES(OVERALL) WITH(Age=MEAN Education_yrs=MEAN GENDER_USE=MEAN BDI_Total=MEAN
   NAART_Total=MEAN ChooseTrainingnErr=MEAN VO2=MEAN)
  /EMMEANS=TABLES(BMI4grp) WITH(Age=MEAN Education_yrs=MEAN GENDER_USE=MEAN BDI_Total=MEAN
   NAART_Total=MEAN ChooseTrainingnErr=MEAN VO2=MEAN) COMPARE ADJ(LSD)
  /EMMEANS=TABLES(APOE_USE) WITH(Age=MEAN Education_yrs=MEAN GENDER_USE=MEAN BDI_Total=MEAN
   NAART_Total=MEAN ChooseTrainingnErr=MEAN VO2=MEAN) COMPARE ADJ(LSD)
  /EMMEANS=TABLES(APOE_USE*BMI4grp) WITH(Age=MEAN Education_yrs=MEAN GENDER_USE=MEAN BDI_Total=MEAN
   NAART_Total=MEAN ChooseTrainingnErr=MEAN VO2=MEAN)
  /PRINT ETASQ DESCRIPTIVE PARAMETER HOMOGENEITY OPOWER
  /CRITERIA=ALPHA(.05)
  /DESIGN=BMI4grp APOE_USE Age Education_yrs GENDER_USE BDI_Total NAART_Total ChooseTrainingnErr VO2
   APOE_USE*BMI4grp.

*************************STRUCTURAL OUTCOMES*************************************
   ******Add intracranial volume as additional covariate*******
   *******May 18, 2023*******

*******1) Left Anterolateral entorhinal cortex**********

UNIANOVA   VLeftaLEC BY BMI4grp APOE_USE WITH Age Education_yrs GENDER_USE BDI_Total VO2
   NAART_Total ICV
  /CONTRAST(BMI4grp)=Repeated
  /CONTRAST(APOE_USE)=Simple(1)
  /METHOD=SSTYPE(3)
  /INTERCEPT=INCLUDE
  /PLOT=PROFILE(BMI4grp*APOE_USE BMI4grp) TYPE=LINE ERRORBAR=SE(2) MEANREFERENCE=NO YAXIS=AUTO

```
  /EMMEANS=TABLES(OVERALL) WITH(Age=MEAN Education_yrs=MEAN GENDER_USE=MEAN
BDI_Total=MEAN
    NAART_Total=MEAN VO2=MEAN ICV=MEAN)
  /EMMEANS=TABLES(BMI4grp) WITH(Age=MEAN Education_yrs=MEAN GENDER_USE=MEAN
BDI_Total=MEAN
    NAART_Total=MEAN VO2=MEAN  ICV=MEAN) COMPARE ADJ(LSD)
  /EMMEANS=TABLES(APOE_USE) WITH(Age=MEAN Education_yrs=MEAN GENDER_USE=MEAN
BDI_Total=MEAN
    NAART_Total=MEAN VO2=MEAN  ICV=MEAN) COMPARE ADJ(LSD)
  /EMMEANS=TABLES(APOE_USE*BMI4grp) WITH(Age=MEAN Education_yrs=MEAN
GENDER_USE=MEAN BDI_Total=MEAN
    NAART_Total=MEAN VO2=MEAN  ICV=MEAN)
  /PRINT ETASQ DESCRIPTIVE PARAMETER HOMOGENEITY OPOWER
  /CRITERIA=ALPHA(.05)
  /DESIGN=BMI4grp APOE_USE Age Education_yrs GENDER_USE BDI_Total NAART_Total VO2
ICV
    APOE_USE*BMI4grp.

UNIANOVA   SALeftaLEC BY BMI4grp APOE_USE WITH Age Education_yrs GENDER_USE
BDI_Total VO2  ICV
    NAART_Total
  /CONTRAST(BMI4grp)=Repeated
  /CONTRAST(APOE_USE)=Simple(1)
  /METHOD=SSTYPE(3)
  /INTERCEPT=INCLUDE
  /PLOT=PROFILE(BMI4grp*APOE_USE BMI4grp) TYPE=LINE ERRORBAR=SE(2)
MEANREFERENCE=NO YAXIS=AUTO
  /EMMEANS=TABLES(OVERALL) WITH(Age=MEAN Education_yrs=MEAN GENDER_USE=MEAN
BDI_Total=MEAN
    NAART_Total=MEAN VO2=MEAN  ICV=MEAN)
  /EMMEANS=TABLES(BMI4grp) WITH(Age=MEAN Education_yrs=MEAN GENDER_USE=MEAN
BDI_Total=MEAN
    NAART_Total=MEAN VO2=MEAN  ICV=MEAN) COMPARE ADJ(LSD)
  /EMMEANS=TABLES(APOE_USE) WITH(Age=MEAN Education_yrs=MEAN GENDER_USE=MEAN
BDI_Total=MEAN
    NAART_Total=MEAN VO2=MEAN  ICV=MEAN) COMPARE ADJ(LSD)
  /EMMEANS=TABLES(APOE_USE*BMI4grp) WITH(Age=MEAN Education_yrs=MEAN
GENDER_USE=MEAN BDI_Total=MEAN
    NAART_Total=MEAN VO2=MEAN  ICV=MEAN)
  /PRINT ETASQ DESCRIPTIVE PARAMETER HOMOGENEITY OPOWER
  /CRITERIA=ALPHA(.05)
  /DESIGN=BMI4grp APOE_USE Age Education_yrs GENDER_USE BDI_Total NAART_Total VO2
ICV
    APOE_USE*BMI4grp.
```

*********2) Right anterolateral entorhinal cortex***************

```
UNIANOVA  VRightaLEC BY BMI4grp APOE_USE WITH Age Education_yrs GENDER_USE
BDI_Total VO2 ICV
    NAART_Total
  /CONTRAST(BMI4grp)=Repeated
  /CONTRAST(APOE_USE)=Simple(1)
  /METHOD=SSTYPE(3)
  /INTERCEPT=INCLUDE
  /PLOT=PROFILE(BMI4grp*APOE_USE BMI4grp) TYPE=LINE ERRORBAR=SE(2)
MEANREFERENCE=NO YAXIS=AUTO
  /EMMEANS=TABLES(OVERALL) WITH(Age=MEAN Education_yrs=MEAN GENDER_USE=MEAN
BDI_Total=MEAN
    NAART_Total=MEAN VO2=MEAN  ICV=MEAN)
  /EMMEANS=TABLES(BMI4grp) WITH(Age=MEAN Education_yrs=MEAN GENDER_USE=MEAN
BDI_Total=MEAN
    NAART_Total=MEAN VO2=MEAN  ICV=MEAN) COMPARE ADJ(LSD)
  /EMMEANS=TABLES(APOE_USE) WITH(Age=MEAN Education_yrs=MEAN GENDER_USE=MEAN
BDI_Total=MEAN
    NAART_Total=MEAN VO2=MEAN  ICV=MEAN) COMPARE ADJ(LSD)
  /EMMEANS=TABLES(APOE_USE*BMI4grp) WITH(Age=MEAN Education_yrs=MEAN
GENDER_USE=MEAN BDI_Total=MEAN
    NAART_Total=MEAN VO2=MEAN  ICV=MEAN)
  /PRINT ETASQ DESCRIPTIVE PARAMETER HOMOGENEITY OPOWER
  /CRITERIA=ALPHA(.05)
  /DESIGN=BMI4grp APOE_USE Age Education_yrs GENDER_USE BDI_Total NAART_Total VO2
ICV
    APOE_USE*BMI4grp.

UNIANOVA  SARightaLEC BY BMI4grp APOE_USE WITH Age Education_yrs GENDER_USE
BDI_Total VO2 ICV
    NAART_Total
  /CONTRAST(BMI4grp)=Repeated
  /CONTRAST(APOE_USE)=Simple(1)
  /METHOD=SSTYPE(3)
  /INTERCEPT=INCLUDE
  /PLOT=PROFILE(BMI4grp*APOE_USE BMI4grp) TYPE=LINE ERRORBAR=SE(2)
MEANREFERENCE=NO YAXIS=AUTO
  /EMMEANS=TABLES(OVERALL) WITH(Age=MEAN Education_yrs=MEAN GENDER_USE=MEAN
BDI_Total=MEAN
    NAART_Total=MEAN VO2=MEAN  ICV=MEAN)
  /EMMEANS=TABLES(BMI4grp) WITH(Age=MEAN Education_yrs=MEAN GENDER_USE=MEAN
BDI_Total=MEAN
```

```
   NAART_Total=MEAN VO2=MEAN  ICV=MEAN) COMPARE ADJ(LSD)
  /EMMEANS=TABLES(APOE_USE) WITH(Age=MEAN Education_yrs=MEAN GENDER_USE=MEAN
BDI_Total=MEAN
   NAART_Total=MEAN VO2=MEAN  ICV=MEAN) COMPARE ADJ(LSD)
  /EMMEANS=TABLES(APOE_USE*BMI4grp) WITH(Age=MEAN Education_yrs=MEAN
GENDER_USE=MEAN BDI_Total=MEAN
   NAART_Total=MEAN VO2=MEAN  ICV=MEAN)
  /PRINT ETASQ DESCRIPTIVE PARAMETER HOMOGENEITY OPOWER
  /CRITERIA=ALPHA(.05)
  /DESIGN=BMI4grp APOE_USE Age Education_yrs GENDER_USE BDI_Total NAART_Total VO2
ICV
   APOE_USE*BMI4grp.
```

**********3) Left posteromedial entorhinal cortex*****************

```
UNIANOVA   VLeftpMEC BY BMI4grp APOE_USE WITH Age Education_yrs GENDER_USE
BDI_Total VO2 ICV
   NAART_Total
  /CONTRAST(BMI4grp)=Repeated
  /CONTRAST(APOE_USE)=Simple(1)
  /METHOD=SSTYPE(3)
  /INTERCEPT=INCLUDE
  /PLOT=PROFILE(BMI4grp*APOE_USE BMI4grp) TYPE=LINE ERRORBAR=SE(2)
MEANREFERENCE=NO YAXIS=AUTO
  /EMMEANS=TABLES(OVERALL) WITH(Age=MEAN Education_yrs=MEAN GENDER_USE=MEAN
BDI_Total=MEAN
   NAART_Total=MEAN VO2=MEAN ICV=MEAN)
  /EMMEANS=TABLES(BMI4grp) WITH(Age=MEAN Education_yrs=MEAN GENDER_USE=MEAN
BDI_Total=MEAN
   NAART_Total=MEAN VO2=MEAN  ICV=MEAN) COMPARE ADJ(LSD)
  /EMMEANS=TABLES(APOE_USE) WITH(Age=MEAN Education_yrs=MEAN GENDER_USE=MEAN
BDI_Total=MEAN
   NAART_Total=MEAN VO2=MEAN  ICV=MEAN) COMPARE ADJ(LSD)
  /EMMEANS=TABLES(APOE_USE*BMI4grp) WITH(Age=MEAN Education_yrs=MEAN
GENDER_USE=MEAN BDI_Total=MEAN
   NAART_Total=MEAN VO2=MEAN  ICV=MEAN)
  /PRINT ETASQ DESCRIPTIVE PARAMETER HOMOGENEITY OPOWER
  /CRITERIA=ALPHA(.05)
  /DESIGN=BMI4grp APOE_USE Age Education_yrs GENDER_USE BDI_Total NAART_Total VO2
ICV
   APOE_USE*BMI4grp.
```

```
UNIANOVA   SALeftpMEC BY BMI4grp APOE_USE WITH Age Education_yrs GENDER_USE
BDI_Total VO2 ICV
    NAART_Total
  /CONTRAST(BMI4grp)=Repeated
  /CONTRAST(APOE_USE)=Simple(1)
  /METHOD=SSTYPE(3)
  /INTERCEPT=INCLUDE
  /PLOT=PROFILE(BMI4grp*APOE_USE BMI4grp) TYPE=LINE ERRORBAR=SE(2)
MEANREFERENCE=NO YAXIS=AUTO
  /EMMEANS=TABLES(OVERALL) WITH(Age=MEAN Education_yrs=MEAN GENDER_USE=MEAN
BDI_Total=MEAN
    NAART_Total=MEAN VO2=MEAN  ICV=MEAN)
  /EMMEANS=TABLES(BMI4grp) WITH(Age=MEAN Education_yrs=MEAN GENDER_USE=MEAN
BDI_Total=MEAN
    NAART_Total=MEAN VO2=MEAN  ICV=MEAN) COMPARE ADJ(LSD)
  /EMMEANS=TABLES(APOE_USE) WITH(Age=MEAN Education_yrs=MEAN GENDER_USE=MEAN
BDI_Total=MEAN
    NAART_Total=MEAN VO2=MEAN  ICV=MEAN) COMPARE ADJ(LSD)
  /EMMEANS=TABLES(APOE_USE*BMI4grp) WITH(Age=MEAN Education_yrs=MEAN
GENDER_USE=MEAN BDI_Total=MEAN
    NAART_Total=MEAN VO2=MEAN  ICV=MEAN)
  /PRINT ETASQ DESCRIPTIVE PARAMETER HOMOGENEITY OPOWER
  /CRITERIA=ALPHA(.05)
  /DESIGN=BMI4grp APOE_USE Age Education_yrs GENDER_USE BDI_Total NAART_Total VO2
ICV
    APOE_USE*BMI4grp.
```

*******************4) Right posteromedial entorhinal cortex********

```
UNIANOVA   VRightpMEC BY BMI4grp APOE_USE WITH Age Education_yrs GENDER_USE
BDI_Total VO2 ICV
    NAART_Total
  /CONTRAST(BMI4grp)=Repeated
  /CONTRAST(APOE_USE)=Simple(1)
  /METHOD=SSTYPE(3)
  /INTERCEPT=INCLUDE
  /PLOT=PROFILE(BMI4grp*APOE_USE BMI4grp) TYPE=LINE ERRORBAR=SE(2)
MEANREFERENCE=NO YAXIS=AUTO
  /EMMEANS=TABLES(OVERALL) WITH(Age=MEAN Education_yrs=MEAN GENDER_USE=MEAN
BDI_Total=MEAN
    NAART_Total=MEAN VO2=MEAN  ICV=MEAN)
  /EMMEANS=TABLES(BMI4grp) WITH(Age=MEAN Education_yrs=MEAN GENDER_USE=MEAN
BDI_Total=MEAN
    NAART_Total=MEAN VO2=MEAN  ICV=MEAN) COMPARE ADJ(LSD)
```

```
  /EMMEANS=TABLES(APOE_USE) WITH(Age=MEAN Education_yrs=MEAN GENDER_USE=MEAN
BDI_Total=MEAN
    NAART_Total=MEAN VO2=MEAN  ICV=MEAN) COMPARE ADJ(LSD)
  /EMMEANS=TABLES(APOE_USE*BMI4grp) WITH(Age=MEAN Education_yrs=MEAN
GENDER_USE=MEAN BDI_Total=MEAN
    NAART_Total=MEAN VO2=MEAN  ICV=MEAN)
  /PRINT ETASQ DESCRIPTIVE PARAMETER HOMOGENEITY OPOWER
  /CRITERIA=ALPHA(.05)
  /DESIGN=BMI4grp APOE_USE Age Education_yrs GENDER_USE BDI_Total NAART_Total VO2
ICV
    APOE_USE*BMI4grp.


UNIANOVA   SARightpMEC BY BMI4grp APOE_USE WITH Age Education_yrs GENDER_USE
BDI_Total VO2 ICV
    NAART_Total
  /CONTRAST(BMI4grp)=Repeated
  /CONTRAST(APOE_USE)=Simple(1)
  /METHOD=SSTYPE(3)
  /INTERCEPT=INCLUDE
  /PLOT=PROFILE(BMI4grp*APOE_USE BMI4grp) TYPE=LINE ERRORBAR=SE(2)
MEANREFERENCE=NO YAXIS=AUTO
  /EMMEANS=TABLES(OVERALL) WITH(Age=MEAN Education_yrs=MEAN GENDER_USE=MEAN
BDI_Total=MEAN
    NAART_Total=MEAN VO2=MEAN  ICV=MEAN)
  /EMMEANS=TABLES(BMI4grp) WITH(Age=MEAN Education_yrs=MEAN GENDER_USE=MEAN
BDI_Total=MEAN
    NAART_Total=MEAN VO2=MEAN  ICV=MEAN) COMPARE ADJ(LSD)
  /EMMEANS=TABLES(APOE_USE) WITH(Age=MEAN Education_yrs=MEAN GENDER_USE=MEAN
BDI_Total=MEAN
    NAART_Total=MEAN VO2=MEAN  ICV=MEAN) COMPARE ADJ(LSD)
  /EMMEANS=TABLES(APOE_USE*BMI4grp) WITH(Age=MEAN Education_yrs=MEAN
GENDER_USE=MEAN BDI_Total=MEAN
    NAART_Total=MEAN VO2=MEAN  ICV=MEAN)
  /PRINT ETASQ DESCRIPTIVE PARAMETER HOMOGENEITY OPOWER
  /CRITERIA=ALPHA(.05)
  /DESIGN=BMI4grp APOE_USE Age Education_yrs GENDER_USE BDI_Total NAART_Total VO2
ICV
    APOE_USE*BMI4grp.



*******5) Left perirhinal cortex*********
```

```
UNIANOVA  VLeftPerirhinalCortex BY BMI4grp APOE_USE WITH Age Education_yrs
GENDER_USE BDI_Total VO2 ICV
    NAART_Total
  /CONTRAST(BMI4grp)=Repeated
  /CONTRAST(APOE_USE)=Simple(1)
  /METHOD=SSTYPE(3)
  /INTERCEPT=INCLUDE
  /PLOT=PROFILE(BMI4grp*APOE_USE BMI4grp) TYPE=LINE ERRORBAR=SE(2)
MEANREFERENCE=NO YAXIS=AUTO
  /EMMEANS=TABLES(OVERALL) WITH(Age=MEAN Education_yrs=MEAN GENDER_USE=MEAN
BDI_Total=MEAN
    NAART_Total=MEAN VO2=MEAN ICV=MEAN)
  /EMMEANS=TABLES(BMI4grp) WITH(Age=MEAN Education_yrs=MEAN GENDER_USE=MEAN
BDI_Total=MEAN
    NAART_Total=MEAN VO2=MEAN ICV=MEAN) COMPARE ADJ(LSD)
  /EMMEANS=TABLES(APOE_USE) WITH(Age=MEAN Education_yrs=MEAN GENDER_USE=MEAN
BDI_Total=MEAN
    NAART_Total=MEAN VO2=MEAN ICV=MEAN) COMPARE ADJ(LSD)
  /EMMEANS=TABLES(APOE_USE*BMI4grp) WITH(Age=MEAN Education_yrs=MEAN
GENDER_USE=MEAN BDI_Total=MEAN
    NAART_Total=MEAN VO2=MEAN ICV=MEAN)
  /PRINT ETASQ DESCRIPTIVE PARAMETER HOMOGENEITY OPOWER
  /CRITERIA=ALPHA(.05)
  /DESIGN=BMI4grp APOE_USE Age Education_yrs GENDER_USE BDI_Total NAART_Total VO2
ICV
    APOE_USE*BMI4grp.

UNIANOVA  SALeftPerirhinalCortex BY BMI4grp APOE_USE WITH Age Education_yrs
GENDER_USE BDI_Total VO2 ICV
    NAART_Total
  /CONTRAST(BMI4grp)=Repeated
  /CONTRAST(APOE_USE)=Simple(1)
  /METHOD=SSTYPE(3)
  /INTERCEPT=INCLUDE
  /PLOT=PROFILE(BMI4grp*APOE_USE BMI4grp) TYPE=LINE ERRORBAR=SE(2)
MEANREFERENCE=NO YAXIS=AUTO
  /EMMEANS=TABLES(OVERALL) WITH(Age=MEAN Education_yrs=MEAN GENDER_USE=MEAN
BDI_Total=MEAN
    NAART_Total=MEAN VO2=MEAN ICV=MEAN)
  /EMMEANS=TABLES(BMI4grp) WITH(Age=MEAN Education_yrs=MEAN GENDER_USE=MEAN
BDI_Total=MEAN
    NAART_Total=MEAN VO2=MEAN ICV=MEAN) COMPARE ADJ(LSD)
  /EMMEANS=TABLES(APOE_USE) WITH(Age=MEAN Education_yrs=MEAN GENDER_USE=MEAN
BDI_Total=MEAN
```

```
    NAART_Total=MEAN VO2=MEAN ICV=MEAN) COMPARE ADJ(LSD)
  /EMMEANS=TABLES(APOE_USE*BMI4grp) WITH(Age=MEAN Education_yrs=MEAN
GENDER_USE=MEAN BDI_Total=MEAN
    NAART_Total=MEAN VO2=MEAN ICV=MEAN)
  /PRINT ETASQ DESCRIPTIVE PARAMETER HOMOGENEITY OPOWER
  /CRITERIA=ALPHA(.05)
  /DESIGN=BMI4grp APOE_USE Age Education_yrs GENDER_USE BDI_Total NAART_Total VO2
ICV
    APOE_USE*BMI4grp.

*******6) Right perirhinal cortex*********

UNIANOVA  VRightPerirhinalCortex BY BMI4grp APOE_USE WITH Age Education_yrs
GENDER_USE BDI_Total VO2 ICV
    NAART_Total
  /CONTRAST(BMI4grp)=Repeated
  /CONTRAST(APOE_USE)=Simple(1)
  /METHOD=SSTYPE(3)
  /INTERCEPT=INCLUDE
  /PLOT=PROFILE(BMI4grp*APOE_USE BMI4grp) TYPE=LINE ERRORBAR=SE(2)
MEANREFERENCE=NO YAXIS=AUTO
  /EMMEANS=TABLES(OVERALL) WITH(Age=MEAN Education_yrs=MEAN GENDER_USE=MEAN
BDI_Total=MEAN
    NAART_Total=MEAN VO2=MEAN ICV=MEAN)
  /EMMEANS=TABLES(BMI4grp) WITH(Age=MEAN Education_yrs=MEAN GENDER_USE=MEAN
BDI_Total=MEAN
    NAART_Total=MEAN VO2=MEAN ICV=MEAN) COMPARE ADJ(LSD)
  /EMMEANS=TABLES(APOE_USE) WITH(Age=MEAN Education_yrs=MEAN GENDER_USE=MEAN
BDI_Total=MEAN
    NAART_Total=MEAN VO2=MEAN ICV=MEAN) COMPARE ADJ(LSD)
  /EMMEANS=TABLES(APOE_USE*BMI4grp) WITH(Age=MEAN Education_yrs=MEAN
GENDER_USE=MEAN BDI_Total=MEAN
    NAART_Total=MEAN VO2=MEAN ICV=MEAN)
  /PRINT ETASQ DESCRIPTIVE PARAMETER HOMOGENEITY OPOWER
  /CRITERIA=ALPHA(.05)
  /DESIGN=BMI4grp APOE_USE Age Education_yrs GENDER_USE BDI_Total NAART_Total VO2
ICV
    APOE_USE*BMI4grp.

UNIANOVA  SARightPerirhinalCortex BY BMI4grp APOE_USE WITH Age Education_yrs
GENDER_USE BDI_Total VO2 ICV
    NAART_Total
  /CONTRAST(BMI4grp)=Repeated
  /CONTRAST(APOE_USE)=Simple(1)
```

```
  /METHOD=SSTYPE(3)
  /INTERCEPT=INCLUDE
  /PLOT=PROFILE(BMI4grp*APOE_USE BMI4grp) TYPE=LINE ERRORBAR=SE(2)
MEANREFERENCE=NO YAXIS=AUTO
  /EMMEANS=TABLES(OVERALL) WITH(Age=MEAN Education_yrs=MEAN GENDER_USE=MEAN
BDI_Total=MEAN
    NAART_Total=MEAN VO2=MEAN ICV=MEAN)
  /EMMEANS=TABLES(BMI4grp) WITH(Age=MEAN Education_yrs=MEAN GENDER_USE=MEAN
BDI_Total=MEAN
    NAART_Total=MEAN VO2=MEAN ICV=MEAN) COMPARE ADJ(LSD)
  /EMMEANS=TABLES(APOE_USE) WITH(Age=MEAN Education_yrs=MEAN GENDER_USE=MEAN
BDI_Total=MEAN
    NAART_Total=MEAN VO2=MEAN ICV=MEAN) COMPARE ADJ(LSD)
  /EMMEANS=TABLES(APOE_USE*BMI4grp) WITH(Age=MEAN Education_yrs=MEAN
GENDER_USE=MEAN BDI_Total=MEAN
    NAART_Total=MEAN VO2=MEAN ICV=MEAN)
  /PRINT ETASQ DESCRIPTIVE PARAMETER HOMOGENEITY OPOWER
  /CRITERIA=ALPHA(.05)
  /DESIGN=BMI4grp APOE_USE Age Education_yrs GENDER_USE BDI_Total NAART_Total VO2
ICV
    APOE_USE*BMI4grp.
```

**********7) Left parahippocampal cortex************

```
UNIANOVA  VLeftParahippocampalCortex BY BMI4grp APOE_USE WITH Age Education_yrs
GENDER_USE BDI_Total VO2 ICV
    NAART_Total
  /CONTRAST(BMI4grp)=Repeated
  /CONTRAST(APOE_USE)=Simple(1)
  /METHOD=SSTYPE(3)
  /INTERCEPT=INCLUDE
  /PLOT=PROFILE(BMI4grp*APOE_USE BMI4grp) TYPE=LINE ERRORBAR=SE(2)
MEANREFERENCE=NO YAXIS=AUTO
  /EMMEANS=TABLES(OVERALL) WITH(Age=MEAN Education_yrs=MEAN GENDER_USE=MEAN
BDI_Total=MEAN
    NAART_Total=MEAN VO2=MEAN ICV=MEAN)
  /EMMEANS=TABLES(BMI4grp) WITH(Age=MEAN Education_yrs=MEAN GENDER_USE=MEAN
BDI_Total=MEAN
    NAART_Total=MEAN VO2=MEAN ICV=MEAN) COMPARE ADJ(LSD)
  /EMMEANS=TABLES(APOE_USE) WITH(Age=MEAN Education_yrs=MEAN GENDER_USE=MEAN
BDI_Total=MEAN
```

```
    NAART_Total=MEAN VO2=MEAN ICV=MEAN) COMPARE ADJ(LSD)
  /EMMEANS=TABLES(APOE_USE*BMI4grp) WITH(Age=MEAN Education_yrs=MEAN
GENDER_USE=MEAN BDI_Total=MEAN
    NAART_Total=MEAN VO2=MEAN ICV=MEAN)
  /PRINT ETASQ DESCRIPTIVE PARAMETER HOMOGENEITY OPOWER
  /CRITERIA=ALPHA(.05)
  /DESIGN=BMI4grp APOE_USE Age Education_yrs GENDER_USE BDI_Total NAART_Total VO2
ICV
    APOE_USE*BMI4grp.


UNIANOVA  SALeftParahippocampalCortex BY BMI4grp APOE_USE WITH Age Education_yrs
GENDER_USE BDI_Total VO2 ICV
    NAART_Total
  /CONTRAST(BMI4grp)=Repeated
  /CONTRAST(APOE_USE)=Simple(1)
  /METHOD=SSTYPE(3)
  /INTERCEPT=INCLUDE
  /PLOT=PROFILE(BMI4grp*APOE_USE BMI4grp) TYPE=LINE ERRORBAR=SE(2)
MEANREFERENCE=NO YAXIS=AUTO
  /EMMEANS=TABLES(OVERALL) WITH(Age=MEAN Education_yrs=MEAN GENDER_USE=MEAN
BDI_Total=MEAN
    NAART_Total=MEAN VO2=MEAN ICV=MEAN)
  /EMMEANS=TABLES(BMI4grp) WITH(Age=MEAN Education_yrs=MEAN GENDER_USE=MEAN
BDI_Total=MEAN
    NAART_Total=MEAN VO2=MEAN ICV=MEAN) COMPARE ADJ(LSD)
  /EMMEANS=TABLES(APOE_USE) WITH(Age=MEAN Education_yrs=MEAN GENDER_USE=MEAN
BDI_Total=MEAN
    NAART_Total=MEAN VO2=MEAN ICV=MEAN) COMPARE ADJ(LSD)
  /EMMEANS=TABLES(APOE_USE*BMI4grp) WITH(Age=MEAN Education_yrs=MEAN
GENDER_USE=MEAN BDI_Total=MEAN
    NAART_Total=MEAN VO2=MEAN ICV=MEAN)
  /PRINT ETASQ DESCRIPTIVE PARAMETER HOMOGENEITY OPOWER
  /CRITERIA=ALPHA(.05)
  /DESIGN=BMI4grp APOE_USE Age Education_yrs GENDER_USE BDI_Total NAART_Total VO2
ICV
    APOE_USE*BMI4grp.
```

**********8) Right parahippocampal cortex************

```
UNIANOVA  VRightParahippocampalCortex BY BMI4grp APOE_USE WITH Age Education_yrs
GENDER_USE BDI_Total VO2 ICV
    NAART_Total
  /CONTRAST(BMI4grp)=Repeated
```

```
  /CONTRAST(APOE_USE)=Simple(1)
  /METHOD=SSTYPE(3)
  /INTERCEPT=INCLUDE
  /PLOT=PROFILE(BMI4grp*APOE_USE BMI4grp) TYPE=LINE ERRORBAR=SE(2)
MEANREFERENCE=NO YAXIS=AUTO
  /EMMEANS=TABLES(OVERALL) WITH(Age=MEAN Education_yrs=MEAN GENDER_USE=MEAN
BDI_Total=MEAN
    NAART_Total=MEAN VO2=MEAN ICV=MEAN)
  /EMMEANS=TABLES(BMI4grp) WITH(Age=MEAN Education_yrs=MEAN GENDER_USE=MEAN
BDI_Total=MEAN
    NAART_Total=MEAN VO2=MEAN ICV=MEAN) COMPARE ADJ(LSD)
  /EMMEANS=TABLES(APOE_USE) WITH(Age=MEAN Education_yrs=MEAN GENDER_USE=MEAN
BDI_Total=MEAN
    NAART_Total=MEAN VO2=MEAN ICV=MEAN) COMPARE ADJ(LSD)
  /EMMEANS=TABLES(APOE_USE*BMI4grp) WITH(Age=MEAN Education_yrs=MEAN
GENDER_USE=MEAN BDI_Total=MEAN
    NAART_Total=MEAN VO2=MEAN ICV=MEAN)
  /PRINT ETASQ DESCRIPTIVE PARAMETER HOMOGENEITY OPOWER
  /CRITERIA=ALPHA(.05)
  /DESIGN=BMI4grp APOE_USE Age Education_yrs GENDER_USE BDI_Total NAART_Total VO2
ICV
    APOE_USE*BMI4grp.

UNIANOVA  SARightParahippocampalCortex BY BMI4grp APOE_USE WITH Age Education_yrs
GENDER_USE BDI_Total VO2 ICV
    NAART_Total
  /CONTRAST(BMI4grp)=Repeated
  /CONTRAST(APOE_USE)=Simple(1)
  /METHOD=SSTYPE(3)
  /INTERCEPT=INCLUDE
  /PLOT=PROFILE(BMI4grp*APOE_USE BMI4grp) TYPE=LINE ERRORBAR=SE(2)
MEANREFERENCE=NO YAXIS=AUTO
  /EMMEANS=TABLES(OVERALL) WITH(Age=MEAN Education_yrs=MEAN GENDER_USE=MEAN
BDI_Total=MEAN
    NAART_Total=MEAN VO2=MEAN ICV=MEAN)
  /EMMEANS=TABLES(BMI4grp) WITH(Age=MEAN Education_yrs=MEAN GENDER_USE=MEAN
BDI_Total=MEAN
    NAART_Total=MEAN VO2=MEAN ICV=MEAN) COMPARE ADJ(LSD)
  /EMMEANS=TABLES(APOE_USE) WITH(Age=MEAN Education_yrs=MEAN GENDER_USE=MEAN
BDI_Total=MEAN
    NAART_Total=MEAN VO2=MEAN ICV=MEAN) COMPARE ADJ(LSD)
  /EMMEANS=TABLES(APOE_USE*BMI4grp) WITH(Age=MEAN Education_yrs=MEAN
GENDER_USE=MEAN BDI_Total=MEAN
    NAART_Total=MEAN VO2=MEAN ICV=MEAN)
```

```
  /PRINT ETASQ DESCRIPTIVE PARAMETER HOMOGENEITY OPOWER
  /CRITERIA=ALPHA(.05)
  /DESIGN=BMI4grp APOE_USE Age Education_yrs GENDER_USE BDI_Total NAART_Total VO2
ICV
    APOE_USE*BMI4grp.
```

********9) Left DG/CA3*********

```
UNIANOVA  VLeftDGCA3 BY BMI4grp APOE_USE WITH Age Education_yrs GENDER_USE
BDI_Total VO2 ICV
    NAART_Total
  /CONTRAST(BMI4grp)=Repeated
  /CONTRAST(APOE_USE)=Simple(1)
  /METHOD=SSTYPE(3)
  /INTERCEPT=INCLUDE
  /PLOT=PROFILE(BMI4grp*APOE_USE BMI4grp) TYPE=LINE ERRORBAR=SE(2)
MEANREFERENCE=NO YAXIS=AUTO
  /EMMEANS=TABLES(OVERALL) WITH(Age=MEAN Education_yrs=MEAN GENDER_USE=MEAN
BDI_Total=MEAN
    NAART_Total=MEAN VO2=MEAN ICV=MEAN)
  /EMMEANS=TABLES(BMI4grp) WITH(Age=MEAN Education_yrs=MEAN GENDER_USE=MEAN
BDI_Total=MEAN
    NAART_Total=MEAN VO2=MEAN ICV=MEAN) COMPARE ADJ(LSD)
  /EMMEANS=TABLES(APOE_USE) WITH(Age=MEAN Education_yrs=MEAN GENDER_USE=MEAN
BDI_Total=MEAN
    NAART_Total=MEAN VO2=MEAN ICV=MEAN) COMPARE ADJ(LSD)
  /EMMEANS=TABLES(APOE_USE*BMI4grp) WITH(Age=MEAN Education_yrs=MEAN
GENDER_USE=MEAN BDI_Total=MEAN
    NAART_Total=MEAN VO2=MEAN ICV=MEAN)
  /PRINT ETASQ DESCRIPTIVE PARAMETER HOMOGENEITY OPOWER
  /CRITERIA=ALPHA(.05)
  /DESIGN=BMI4grp APOE_USE Age Education_yrs GENDER_USE BDI_Total NAART_Total VO2
ICV
    APOE_USE*BMI4grp.

UNIANOVA  SALeftDGCA3  BY BMI4grp APOE_USE WITH Age Education_yrs GENDER_USE
BDI_Total VO2 ICV
    NAART_Total
  /CONTRAST(BMI4grp)=Repeated
  /CONTRAST(APOE_USE)=Simple(1)
  /METHOD=SSTYPE(3)
  /INTERCEPT=INCLUDE
```

```
  /PLOT=PROFILE(BMI4grp*APOE_USE BMI4grp) TYPE=LINE ERRORBAR=SE(2)
MEANREFERENCE=NO YAXIS=AUTO
  /EMMEANS=TABLES(OVERALL) WITH(Age=MEAN Education_yrs=MEAN GENDER_USE=MEAN
BDI_Total=MEAN
    NAART_Total=MEAN VO2=MEAN ICV=MEAN)
  /EMMEANS=TABLES(BMI4grp) WITH(Age=MEAN Education_yrs=MEAN GENDER_USE=MEAN
BDI_Total=MEAN
    NAART_Total=MEAN VO2=MEAN ICV=MEAN) COMPARE ADJ(LSD)
  /EMMEANS=TABLES(APOE_USE) WITH(Age=MEAN Education_yrs=MEAN GENDER_USE=MEAN
BDI_Total=MEAN
    NAART_Total=MEAN VO2=MEAN ICV=MEAN) COMPARE ADJ(LSD)
  /EMMEANS=TABLES(APOE_USE*BMI4grp) WITH(Age=MEAN Education_yrs=MEAN
GENDER_USE=MEAN BDI_Total=MEAN
    NAART_Total=MEAN VO2=MEAN ICV=MEAN)
  /PRINT ETASQ DESCRIPTIVE PARAMETER HOMOGENEITY OPOWER
  /CRITERIA=ALPHA(.05)
  /DESIGN=BMI4grp APOE_USE Age Education_yrs GENDER_USE BDI_Total NAART_Total VO2
ICV
    APOE_USE*BMI4grp.
```

*******10) Right DG/CA3*********

```
UNIANOVA  VRightDGCA3 BY BMI4grp APOE_USE WITH Age Education_yrs GENDER_USE
BDI_Total VO2 ICV
    NAART_Total
  /CONTRAST(BMI4grp)=Repeated
  /CONTRAST(APOE_USE)=Simple(1)
  /METHOD=SSTYPE(3)
  /INTERCEPT=INCLUDE
  /PLOT=PROFILE(BMI4grp*APOE_USE BMI4grp) TYPE=LINE ERRORBAR=SE(2)
MEANREFERENCE=NO YAXIS=AUTO
  /EMMEANS=TABLES(OVERALL) WITH(Age=MEAN Education_yrs=MEAN GENDER_USE=MEAN
BDI_Total=MEAN
    NAART_Total=MEAN VO2=MEAN ICV=MEAN)
  /EMMEANS=TABLES(BMI4grp) WITH(Age=MEAN Education_yrs=MEAN GENDER_USE=MEAN
BDI_Total=MEAN
    NAART_Total=MEAN VO2=MEAN ICV=MEAN) COMPARE ADJ(LSD)
  /EMMEANS=TABLES(APOE_USE) WITH(Age=MEAN Education_yrs=MEAN GENDER_USE=MEAN
BDI_Total=MEAN
    NAART_Total=MEAN VO2=MEAN ICV=MEAN) COMPARE ADJ(LSD)
  /EMMEANS=TABLES(APOE_USE*BMI4grp) WITH(Age=MEAN Education_yrs=MEAN
GENDER_USE=MEAN BDI_Total=MEAN
    NAART_Total=MEAN VO2=MEAN ICV=MEAN)
```

```
  /PRINT ETASQ DESCRIPTIVE PARAMETER HOMOGENEITY OPOWER
  /CRITERIA=ALPHA(.05)
  /DESIGN=BMI4grp APOE_USE Age Education_yrs GENDER_USE BDI_Total NAART_Total VO2
ICV
    APOE_USE*BMI4grp.

UNIANOVA  SARightDGCA3 BY BMI4grp APOE_USE WITH Age Education_yrs GENDER_USE
BDI_Total VO2 ICV
    NAART_Total
  /CONTRAST(BMI4grp)=Repeated
  /CONTRAST(APOE_USE)=Simple(1)
  /METHOD=SSTYPE(3)
  /INTERCEPT=INCLUDE
  /PLOT=PROFILE(BMI4grp*APOE_USE BMI4grp) TYPE=LINE ERRORBAR=SE(2)
MEANREFERENCE=NO YAXIS=AUTO
  /EMMEANS=TABLES(OVERALL) WITH(Age=MEAN Education_yrs=MEAN GENDER_USE=MEAN
BDI_Total=MEAN
    NAART_Total=MEAN VO2=MEAN ICV=MEAN)
  /EMMEANS=TABLES(BMI4grp) WITH(Age=MEAN Education_yrs=MEAN GENDER_USE=MEAN
BDI_Total=MEAN
    NAART_Total=MEAN VO2=MEAN ICV=MEAN) COMPARE ADJ(LSD)
  /EMMEANS=TABLES(APOE_USE) WITH(Age=MEAN Education_yrs=MEAN GENDER_USE=MEAN
BDI_Total=MEAN
    NAART_Total=MEAN VO2=MEAN ICV=MEAN) COMPARE ADJ(LSD)
  /EMMEANS=TABLES(APOE_USE*BMI4grp) WITH(Age=MEAN Education_yrs=MEAN
GENDER_USE=MEAN BDI_Total=MEAN
    NAART_Total=MEAN VO2=MEAN ICV=MEAN)
  /PRINT ETASQ DESCRIPTIVE PARAMETER HOMOGENEITY OPOWER
  /CRITERIA=ALPHA(.05)
  /DESIGN=BMI4grp APOE_USE Age Education_yrs GENDER_USE BDI_Total NAART_Total VO2
ICV
    APOE_USE*BMI4grp.

********11) Left CA1*********

UNIANOVA   VLeftCA1 BY BMI4grp APOE_USE WITH Age Education_yrs GENDER_USE BDI_Total
VO2 ICV
    NAART_Total
  /CONTRAST(BMI4grp)=Repeated
  /CONTRAST(APOE_USE)=Simple(1)
  /METHOD=SSTYPE(3)
  /INTERCEPT=INCLUDE
  /PLOT=PROFILE(BMI4grp*APOE_USE BMI4grp) TYPE=LINE ERRORBAR=SE(2)
MEANREFERENCE=NO YAXIS=AUTO
```

```
  /EMMEANS=TABLES(OVERALL) WITH(Age=MEAN Education_yrs=MEAN GENDER_USE=MEAN
BDI_Total=MEAN
    NAART_Total=MEAN VO2=MEAN ICV=MEAN)
  /EMMEANS=TABLES(BMI4grp) WITH(Age=MEAN Education_yrs=MEAN GENDER_USE=MEAN
BDI_Total=MEAN
    NAART_Total=MEAN VO2=MEAN ICV=MEAN) COMPARE ADJ(LSD)
  /EMMEANS=TABLES(APOE_USE) WITH(Age=MEAN Education_yrs=MEAN GENDER_USE=MEAN
BDI_Total=MEAN
    NAART_Total=MEAN VO2=MEAN ICV=MEAN) COMPARE ADJ(LSD)
  /EMMEANS=TABLES(APOE_USE*BMI4grp) WITH(Age=MEAN Education_yrs=MEAN
GENDER_USE=MEAN BDI_Total=MEAN
    NAART_Total=MEAN VO2=MEAN ICV=MEAN)
  /PRINT ETASQ DESCRIPTIVE PARAMETER HOMOGENEITY OPOWER
  /CRITERIA=ALPHA(.05)
  /DESIGN=BMI4grp APOE_USE Age Education_yrs GENDER_USE BDI_Total NAART_Total VO2
ICV
    APOE_USE*BMI4grp.
```

******significant at p<.05, followed up with separate post-hoc one-way ANCOVAs fixed at APOE low and high risk******

*****low risk APOE filter*****

```
USE ALL.
COMPUTE filter_$=(APOE_USE=0).
VARIABLE LABELS filter_$ 'APOE_USE=0 (FILTER)'.
VALUE LABELS filter_$ 0 'Not Selected' 1 'Selected'.
FORMATS filter_$ (f1.0).
FILTER BY filter_$.
EXECUTE.

UNIANOVA   VLeftCA1 BY BMI4grp WITH Age Education_yrs GENDER_USE BDI_Total
NAART_Total VO2 ICV
  /CONTRAST(BMI4grp)=Repeated
  /METHOD=SSTYPE(3)
  /INTERCEPT=INCLUDE
  /PLOT=PROFILE(BMI4grp) TYPE=LINE ERRORBAR=SE(2) MEANREFERENCE=NO YAXIS=AUTO
  /EMMEANS=TABLES(OVERALL) WITH(Age=MEAN Education_yrs=MEAN GENDER_USE=MEAN
BDI_Total=MEAN
    NAART_Total=MEAN VO2=MEAN ICV=MEAN)
  /EMMEANS=TABLES(BMI4grp) WITH(Age=MEAN Education_yrs=MEAN GENDER_USE=MEAN
BDI_Total=MEAN
    NAART_Total=MEAN VO2=MEAN  ICV=MEAN) COMPARE ADJ(LSD)
  /PRINT ETASQ DESCRIPTIVE PARAMETER HOMOGENEITY OPOWER
```

```
  /CRITERIA=ALPHA(.05)
  /DESIGN=BMI4grp Age Education_yrs GENDER_USE BDI_Total NAART_Total VO2 ICV.

*****high risk APOE filter*****

USE ALL.
COMPUTE filter_$=(APOE_USE=1).
VARIABLE LABELS filter_$ 'APOE_USE=1 (FILTER)'.
VALUE LABELS filter_$ 0 'Not Selected' 1 'Selected'.
FORMATS filter_$ (f1.0).
FILTER BY filter_$.
EXECUTE.

UNIANOVA VLeftCA1 BY BMI4grp WITH Age Education_yrs GENDER_USE BDI_Total NAART_Total
VO2 ICV
  /CONTRAST(BMI4grp)=Repeated
  /METHOD=SSTYPE(3)
  /INTERCEPT=INCLUDE
  /PLOT=PROFILE(BMI4grp) TYPE=LINE ERRORBAR=SE(2) MEANREFERENCE=NO YAXIS=AUTO
  /EMMEANS=TABLES(OVERALL) WITH(Age=MEAN Education_yrs=MEAN GENDER_USE=MEAN
BDI_Total=MEAN
    NAART_Total=MEAN VO2=MEAN  ICV=MEAN)
  /EMMEANS=TABLES(BMI4grp) WITH(Age=MEAN Education_yrs=MEAN GENDER_USE=MEAN
BDI_Total=MEAN
    NAART_Total=MEAN VO2=MEAN  ICV=MEAN) COMPARE ADJ(LSD)
  /PRINT ETASQ DESCRIPTIVE PARAMETER HOMOGENEITY OPOWER
  /CRITERIA=ALPHA(.05)
  /DESIGN=BMI4grp Age Education_yrs GENDER_USE BDI_Total NAART_Total VO2 ICV.

***left CA1 surface area**

UNIANOVA   SALeftCA1 BY BMI4grp APOE_USE WITH Age Education_yrs GENDER_USE BDI_Total
VO2 ICV
    NAART_Total
  /CONTRAST(BMI4grp)=Repeated
  /CONTRAST(APOE_USE)=Simple(1)
  /METHOD=SSTYPE(3)
  /INTERCEPT=INCLUDE
  /PLOT=PROFILE(BMI4grp*APOE_USE BMI4grp) TYPE=LINE ERRORBAR=SE(2)
MEANREFERENCE=NO YAXIS=AUTO
  /EMMEANS=TABLES(OVERALL) WITH(Age=MEAN Education_yrs=MEAN GENDER_USE=MEAN
BDI_Total=MEAN
    NAART_Total=MEAN VO2=MEAN ICV=MEAN)
```

```
  /EMMEANS=TABLES(BMI4grp) WITH(Age=MEAN Education_yrs=MEAN GENDER_USE=MEAN
BDI_Total=MEAN
    NAART_Total=MEAN VO2=MEAN ICV=MEAN) COMPARE ADJ(LSD)
  /EMMEANS=TABLES(APOE_USE) WITH(Age=MEAN Education_yrs=MEAN GENDER_USE=MEAN
BDI_Total=MEAN
    NAART_Total=MEAN VO2=MEAN ICV=MEAN) COMPARE ADJ(LSD)
  /EMMEANS=TABLES(APOE_USE*BMI4grp) WITH(Age=MEAN Education_yrs=MEAN
GENDER_USE=MEAN BDI_Total=MEAN
    NAART_Total=MEAN VO2=MEAN ICV=MEAN)
  /PRINT ETASQ DESCRIPTIVE PARAMETER HOMOGENEITY OPOWER
  /CRITERIA=ALPHA(.05)
  /DESIGN=BMI4grp APOE_USE Age Education_yrs GENDER_USE BDI_Total NAART_Total VO2
ICV
    APOE_USE*BMI4grp.

******significant at p<.05, followed up with separate post-hoc one-way ANCOVAs fixed at APOE
low and high risk******

*****low risk APOE filter*****

USE ALL.
COMPUTE filter_$=(APOE_USE=0).
VARIABLE LABELS filter_$ 'APOE_USE=0 (FILTER)'.
VALUE LABELS filter_$ 0 'Not Selected' 1 'Selected'.
FORMATS filter_$ (f1.0).
FILTER BY filter_$.
EXECUTE.

UNIANOVA  SALeftCA1 BY BMI4grp WITH Age Education_yrs GENDER_USE BDI_Total
NAART_Total VO2 ICV
  /CONTRAST(BMI4grp)=Repeated
  /METHOD=SSTYPE(3)
  /INTERCEPT=INCLUDE
  /PLOT=PROFILE(BMI4grp) TYPE=LINE ERRORBAR=SE(2) MEANREFERENCE=NO YAXIS=AUTO
  /EMMEANS=TABLES(OVERALL) WITH(Age=MEAN Education_yrs=MEAN GENDER_USE=MEAN
BDI_Total=MEAN
    NAART_Total=MEAN VO2=MEAN ICV=MEAN)
  /EMMEANS=TABLES(BMI4grp) WITH(Age=MEAN Education_yrs=MEAN GENDER_USE=MEAN
BDI_Total=MEAN
    NAART_Total=MEAN VO2=MEAN ICV=MEAN) COMPARE ADJ(LSD)
  /PRINT ETASQ DESCRIPTIVE PARAMETER HOMOGENEITY OPOWER
  /CRITERIA=ALPHA(.05)
  /DESIGN=BMI4grp Age Education_yrs GENDER_USE BDI_Total NAART_Total VO2 ICV.
```

```
*****high risk APOE filter*****

USE ALL.
COMPUTE filter_$=(APOE_USE=1).
VARIABLE LABELS filter_$ 'APOE_USE=1 (FILTER)'.
VALUE LABELS filter_$ 0 'Not Selected' 1 'Selected'.
FORMATS filter_$ (f1.0).
FILTER BY filter_$.
EXECUTE.

UNIANOVA SALeftCA1 BY BMI4grp WITH Age Education_yrs GENDER_USE BDI_Total
NAART_Total VO2 ICV
  /CONTRAST(BMI4grp)=Repeated
  /METHOD=SSTYPE(3)
  /INTERCEPT=INCLUDE
  /PLOT=PROFILE(BMI4grp) TYPE=LINE ERRORBAR=SE(2) MEANREFERENCE=NO YAXIS=AUTO
  /EMMEANS=TABLES(OVERALL) WITH(Age=MEAN Education_yrs=MEAN GENDER_USE=MEAN
BDI_Total=MEAN
    NAART_Total=MEAN VO2=MEAN ICV=MEAN)
  /EMMEANS=TABLES(BMI4grp) WITH(Age=MEAN Education_yrs=MEAN GENDER_USE=MEAN
BDI_Total=MEAN
    NAART_Total=MEAN VO2=MEAN ICV=MEAN) COMPARE ADJ(LSD)
  /PRINT ETASQ DESCRIPTIVE PARAMETER HOMOGENEITY OPOWER
  /CRITERIA=ALPHA(.05)
  /DESIGN=BMI4grp Age Education_yrs GENDER_USE BDI_Total NAART_Total VO2 ICV.

FILTER OFF.
USE ALL.
EXECUTE.

********12) Right CA1*********

UNIANOVA  VRightCA1 BY BMI4grp APOE_USE WITH Age Education_yrs GENDER_USE BDI_Total
VO2 ICV
    NAART_Total
  /CONTRAST(BMI4grp)=Repeated
  /CONTRAST(APOE_USE)=Simple(1)
  /METHOD=SSTYPE(3)
  /INTERCEPT=INCLUDE
  /PLOT=PROFILE(BMI4grp*APOE_USE BMI4grp) TYPE=LINE ERRORBAR=SE(2)
MEANREFERENCE=NO YAXIS=AUTO
  /EMMEANS=TABLES(OVERALL) WITH(Age=MEAN Education_yrs=MEAN GENDER_USE=MEAN
BDI_Total=MEAN
    NAART_Total=MEAN VO2=MEAN ICV=MEAN)
```

```
  /EMMEANS=TABLES(BMI4grp) WITH(Age=MEAN Education_yrs=MEAN GENDER_USE=MEAN
BDI_Total=MEAN
    NAART_Total=MEAN VO2=MEAN ICV=MEAN) COMPARE ADJ(LSD)
  /EMMEANS=TABLES(APOE_USE) WITH(Age=MEAN Education_yrs=MEAN GENDER_USE=MEAN
BDI_Total=MEAN
    NAART_Total=MEAN VO2=MEAN ICV=MEAN) COMPARE ADJ(LSD)
  /EMMEANS=TABLES(APOE_USE*BMI4grp) WITH(Age=MEAN Education_yrs=MEAN
GENDER_USE=MEAN BDI_Total=MEAN
    NAART_Total=MEAN VO2=MEAN ICV=MEAN)
  /PRINT ETASQ DESCRIPTIVE PARAMETER HOMOGENEITY OPOWER
  /CRITERIA=ALPHA(.05)
  /DESIGN=BMI4grp APOE_USE Age Education_yrs GENDER_USE BDI_Total NAART_Total VO2
ICV
    APOE_USE*BMI4grp.


UNIANOVA  SARightCA1 BY BMI4grp APOE_USE WITH Age Education_yrs GENDER_USE
BDI_Total VO2 ICV
    NAART_Total
  /CONTRAST(BMI4grp)=Repeated
  /CONTRAST(APOE_USE)=Simple(1)
  /METHOD=SSTYPE(3)
  /INTERCEPT=INCLUDE
  /PLOT=PROFILE(BMI4grp*APOE_USE BMI4grp) TYPE=LINE ERRORBAR=SE(2)
MEANREFERENCE=NO YAXIS=AUTO
  /EMMEANS=TABLES(OVERALL) WITH(Age=MEAN Education_yrs=MEAN GENDER_USE=MEAN
BDI_Total=MEAN
    NAART_Total=MEAN VO2=MEAN ICV=MEAN)
  /EMMEANS=TABLES(BMI4grp) WITH(Age=MEAN Education_yrs=MEAN GENDER_USE=MEAN
BDI_Total=MEAN
    NAART_Total=MEAN VO2=MEAN ICV=MEAN) COMPARE ADJ(LSD)
  /EMMEANS=TABLES(APOE_USE) WITH(Age=MEAN Education_yrs=MEAN GENDER_USE=MEAN
BDI_Total=MEAN
    NAART_Total=MEAN VO2=MEAN ICV=MEAN) COMPARE ADJ(LSD)
  /EMMEANS=TABLES(APOE_USE*BMI4grp) WITH(Age=MEAN Education_yrs=MEAN
GENDER_USE=MEAN BDI_Total=MEAN
    NAART_Total=MEAN VO2=MEAN ICV=MEAN)
  /PRINT ETASQ DESCRIPTIVE PARAMETER HOMOGENEITY OPOWER
  /CRITERIA=ALPHA(.05)
  /DESIGN=BMI4grp APOE_USE Age Education_yrs GENDER_USE BDI_Total NAART_Total VO2
ICV
    APOE_USE*BMI4grp.

********13) Left subiculum*********
```

```
UNIANOVA  VLeftSubiculum BY BMI4grp APOE_USE WITH Age Education_yrs GENDER_USE
BDI_Total VO2 ICV
    NAART_Total
  /CONTRAST(BMI4grp)=Repeated
  /CONTRAST(APOE_USE)=Simple(1)
  /METHOD=SSTYPE(3)
  /INTERCEPT=INCLUDE
  /PLOT=PROFILE(BMI4grp*APOE_USE BMI4grp) TYPE=LINE ERRORBAR=SE(2)
MEANREFERENCE=NO YAXIS=AUTO
  /EMMEANS=TABLES(OVERALL) WITH(Age=MEAN Education_yrs=MEAN GENDER_USE=MEAN
BDI_Total=MEAN
    NAART_Total=MEAN VO2=MEAN ICV=MEAN)
  /EMMEANS=TABLES(BMI4grp) WITH(Age=MEAN Education_yrs=MEAN GENDER_USE=MEAN
BDI_Total=MEAN
    NAART_Total=MEAN VO2=MEAN ICV=MEAN) COMPARE ADJ(LSD)
  /EMMEANS=TABLES(APOE_USE) WITH(Age=MEAN Education_yrs=MEAN GENDER_USE=MEAN
BDI_Total=MEAN
    NAART_Total=MEAN VO2=MEAN ICV=MEAN) COMPARE ADJ(LSD)
  /EMMEANS=TABLES(APOE_USE*BMI4grp) WITH(Age=MEAN Education_yrs=MEAN
GENDER_USE=MEAN BDI_Total=MEAN
    NAART_Total=MEAN VO2=MEAN ICV=MEAN)
  /PRINT ETASQ DESCRIPTIVE PARAMETER HOMOGENEITY OPOWER
  /CRITERIA=ALPHA(.05)
  /DESIGN=BMI4grp APOE_USE Age Education_yrs GENDER_USE BDI_Total NAART_Total VO2
ICV
    APOE_USE*BMI4grp.

UNIANOVA  SALeftSubiculum BY BMI4grp APOE_USE WITH Age Education_yrs GENDER_USE
BDI_Total VO2 ICV
    NAART_Total
  /CONTRAST(BMI4grp)=Repeated
  /CONTRAST(APOE_USE)=Simple(1)
  /METHOD=SSTYPE(3)
  /INTERCEPT=INCLUDE
  /PLOT=PROFILE(BMI4grp*APOE_USE BMI4grp) TYPE=LINE ERRORBAR=SE(2)
MEANREFERENCE=NO YAXIS=AUTO
  /EMMEANS=TABLES(OVERALL) WITH(Age=MEAN Education_yrs=MEAN GENDER_USE=MEAN
BDI_Total=MEAN
    NAART_Total=MEAN VO2=MEAN ICV=MEAN)
  /EMMEANS=TABLES(BMI4grp) WITH(Age=MEAN Education_yrs=MEAN GENDER_USE=MEAN
BDI_Total=MEAN
    NAART_Total=MEAN VO2=MEAN ICV=MEAN) COMPARE ADJ(LSD)
```

```
  /EMMEANS=TABLES(APOE_USE) WITH(Age=MEAN Education_yrs=MEAN GENDER_USE=MEAN
BDI_Total=MEAN
    NAART_Total=MEAN VO2=MEAN ICV=MEAN) COMPARE ADJ(LSD)
  /EMMEANS=TABLES(APOE_USE*BMI4grp) WITH(Age=MEAN Education_yrs=MEAN
GENDER_USE=MEAN BDI_Total=MEAN
    NAART_Total=MEAN VO2=MEAN ICV=MEAN)
  /PRINT ETASQ DESCRIPTIVE PARAMETER HOMOGENEITY OPOWER
  /CRITERIA=ALPHA(.05)
  /DESIGN=BMI4grp APOE_USE Age Education_yrs GENDER_USE BDI_Total NAART_Total VO2
ICV
    APOE_USE*BMI4grp.
```

********14) Right subiculum*********

```
UNIANOVA  VRightSubiculum BY BMI4grp APOE_USE WITH Age Education_yrs GENDER_USE
BDI_Total VO2 ICV
    NAART_Total
  /CONTRAST(BMI4grp)=Repeated
  /CONTRAST(APOE_USE)=Simple(1)
  /METHOD=SSTYPE(3)
  /INTERCEPT=INCLUDE
  /PLOT=PROFILE(BMI4grp*APOE_USE BMI4grp) TYPE=LINE ERRORBAR=SE(2)
MEANREFERENCE=NO YAXIS=AUTO
  /EMMEANS=TABLES(OVERALL) WITH(Age=MEAN Education_yrs=MEAN GENDER_USE=MEAN
BDI_Total=MEAN
    NAART_Total=MEAN VO2=MEAN ICV=MEAN)
  /EMMEANS=TABLES(BMI4grp) WITH(Age=MEAN Education_yrs=MEAN GENDER_USE=MEAN
BDI_Total=MEAN
    NAART_Total=MEAN VO2=MEAN ICV=MEAN) COMPARE ADJ(LSD)
  /EMMEANS=TABLES(APOE_USE) WITH(Age=MEAN Education_yrs=MEAN GENDER_USE=MEAN
BDI_Total=MEAN
    NAART_Total=MEAN VO2=MEAN ICV=MEAN) COMPARE ADJ(LSD)
  /EMMEANS=TABLES(APOE_USE*BMI4grp) WITH(Age=MEAN Education_yrs=MEAN
GENDER_USE=MEAN BDI_Total=MEAN
    NAART_Total=MEAN VO2=MEAN ICV=MEAN)
  /PRINT ETASQ DESCRIPTIVE PARAMETER HOMOGENEITY OPOWER
  /CRITERIA=ALPHA(.05)
  /DESIGN=BMI4grp APOE_USE Age Education_yrs GENDER_USE BDI_Total NAART_Total VO2
ICV
    APOE_USE*BMI4grp.
```

```
UNIANOVA   SARightSubiculum BY BMI4grp APOE_USE WITH Age Education_yrs GENDER_USE
BDI_Total VO2 ICV
    NAART_Total
  /CONTRAST(BMI4grp)=Repeated
  /CONTRAST(APOE_USE)=Simple(1)
  /METHOD=SSTYPE(3)
  /INTERCEPT=INCLUDE
  /PLOT=PROFILE(BMI4grp*APOE_USE BMI4grp) TYPE=LINE ERRORBAR=SE(2)
MEANREFERENCE=NO YAXIS=AUTO
  /EMMEANS=TABLES(OVERALL) WITH(Age=MEAN Education_yrs=MEAN GENDER_USE=MEAN
BDI_Total=MEAN
    NAART_Total=MEAN VO2=MEAN ICV=MEAN)
  /EMMEANS=TABLES(BMI4grp) WITH(Age=MEAN Education_yrs=MEAN GENDER_USE=MEAN
BDI_Total=MEAN
    NAART_Total=MEAN VO2=MEAN ICV=MEAN) COMPARE ADJ(LSD)
  /EMMEANS=TABLES(APOE_USE) WITH(Age=MEAN Education_yrs=MEAN GENDER_USE=MEAN
BDI_Total=MEAN
    NAART_Total=MEAN VO2=MEAN ICV=MEAN) COMPARE ADJ(LSD)
  /EMMEANS=TABLES(APOE_USE*BMI4grp) WITH(Age=MEAN Education_yrs=MEAN
GENDER_USE=MEAN BDI_Total=MEAN
    NAART_Total=MEAN VO2=MEAN ICV=MEAN)
  /PRINT ETASQ DESCRIPTIVE PARAMETER HOMOGENEITY OPOWER
  /CRITERIA=ALPHA(.05)
  /DESIGN=BMI4grp APOE_USE Age Education_yrs GENDER_USE BDI_Total NAART_Total VO2
ICV
    APOE_USE*BMI4grp.
```

******Revise & Resubmit Due July 22, 2023******

```
DESCRIPTIVES VARIABLES=
    VLeftaLEC SALeftaLEC
    VRightaLEC SARightaLEC
    VLeftpMEC SALeftpMEC
    VRightpMEC SARightpMEC
    VLeftPerirhinalCortex SALeftPerirhinalCortex
    VRightPerirhinalCortex SARightPerirhinalCortex
    VLeftParahippocampalCortex SALeftParahippocampalCortex
    VRightParahippocampalCortex SARightParahippocampalCortex
    VLeftDGCA3 SALeftDGCA3
    VRightDGCA3 SARightDGCA3
    VLeftCA1 SALeftCA1
    VRightCA1 SARightCA1
    VLeftSubiculum SALeftSubiculum
```

```
   VRightSubiculum SARightSubiculum
  /STATISTICS=MEAN STDDEV MIN MAX.


DESCRIPTIVES VARIABLES=
   VLeftaLEC SALeftaLEC
   VRightaLEC SARightaLEC
   VLeftpMEC SALeftpMEC
   VRightpMEC SARightpMEC
   VLeftPerirhinalCortex SALeftPerirhinalCortex
   VRightPerirhinalCortex SARightPerirhinalCortex
   VLeftParahippocampalCortex SALeftParahippocampalCortex
   VRightParahippocampalCortex SARightParahippocampalCortex
   VLeftDGCA3 SALeftDGCA3
   VRightDGCA3 SARightDGCA3
   VLeftCA1 SALeftCA1
   VRightCA1 SARightCA1
   VLeftSubiculum SALeftSubiculum
   VRightSubiculum SARightSubiculum
  /STATISTICS=MEAN STDDEV MIN MAX.

DATASET ACTIVATE DataSet4.
SORT CASES  BY BMIgroup.
SPLIT FILE LAYERED BY BMIgroup.


DESCRIPTIVES VARIABLES=
   VLeftaLEC SALeftaLEC
   VRightaLEC SARightaLEC
   VLeftpMEC SALeftpMEC
   VRightpMEC SARightpMEC
   VLeftPerirhinalCortex SALeftPerirhinalCortex
   VRightPerirhinalCortex SARightPerirhinalCortex
   VLeftParahippocampalCortex SALeftParahippocampalCortex
   VRightParahippocampalCortex SARightParahippocampalCortex
   VLeftDGCA3 SALeftDGCA3
   VRightDGCA3 SARightDGCA3
   VLeftCA1 SALeftCA1
   VRightCA1 SARightCA1
   VLeftSubiculum SALeftSubiculum
   VRightSubiculum SARightSubiculum
    ICV
  /STATISTICS=MEAN STDDEV MIN MAX.
```

```
SORT CASES BY Subject.
ALTER TYPE Subject (A10).
DATASET ACTIVATE DataSet4.
SORT CASES BY SUBJECT.
DATASET ACTIVATE DataSet3.
MATCH FILES /FILE=*
  /FILE='DataSet4'
  /BY SUBJECT.
EXECUTE.
```
